# Dynamics of MBD2 deposition across methylated DNA regions during malignant transformation of human mammary epithelial cells

**DOI:** 10.1093/nar/gkv508

**Published:** 2015-05-24

**Authors:** Guillaume Devailly, Mélodie Grandin, Laury Perriaud, Pauline Mathot, Jean-Guy Delcros, Yannick Bidet, Anne-Pierre Morel, Jean-Yves Bignon, Alain Puisieux, Patrick Mehlen, Robert Dante

**Affiliations:** 1 Dependence Receptors, Cancer and Development Laboratory - Equipe labellisée ‘La Ligue’, LabEx DEVweCAN, Centre de Recherche en Cancérologie de Lyon (CRCL), INSERM U1052-CNRS UMR5286, Université de Lyon, Centre Léon Bérard, 69008 Lyon, France; 2 Institut Curie and INSERM U612, Centre Universitaire, 91405, Orsay, France; 3 Laboratoire d'Oncologie Moléculaire, Centre Jean Perrin, 63011 Clermont-Ferrand, France; 4 EMT and cancer cell plasticity Laboratory - Equipe labellisée ‘La Ligue’, LabEx DEVweCAN, CRCL, INSERM U1052-CNRS UMR5286, Université de Lyon, Centre Léon Bérard, 69008 Lyon, France

## Abstract

DNA methylation is thought to induce transcriptional silencing through the combination of two mechanisms: the repulsion of transcriptional activators unable to bind their target sites when methylated, and the recruitment of transcriptional repressors with specific affinity for methylated DNA. The Methyl CpG Binding Domain proteins MeCP2, MBD1 and MBD2 belong to the latter category. Here, we present MBD2 ChIPseq data obtained from the endogenous MBD2 in an isogenic cellular model of oncogenic transformation of human mammary cells. In immortalized (HMEC-hTERT) or transformed (HMLER) cells, MBD2 was found in a large proportion of methylated regions and associated with transcriptional silencing. A redistribution of MBD2 on methylated DNA occurred during oncogenic transformation, frequently independently of local DNA methylation changes. Genes downregulated during HMEC-hTERT transformation preferentially gained MBD2 on their promoter. Furthermore, depletion of MBD2 induced an upregulation of MBD2-bound genes methylated at their promoter regions, in HMLER cells. Among the 3,160 genes downregulated in transformed cells, 380 genes were methylated at their promoter regions in both cell lines, specifically associated by MBD2 in HMLER cells, and upregulated upon MBD2 depletion in HMLER. The transcriptional MBD2-dependent downregulation occurring during oncogenic transformation was also observed in two additional models of mammary cell transformation. Thus, the dynamics of MBD2 deposition across methylated DNA regions was associated with the oncogenic transformation of human mammary cells.

## INTRODUCTION

In vertebrates, DNA methylation at transcriptional start sites (TSSs) is an epigenetic modification associated with the downregulation of gene transcription (1). This epigenetic modification has been extensively studied during cell differentiation and neoplastic transformation, since DNA methylation changes are associated with these biological processes and may be involved in the control of gene expression (2–4). Although DNA methylation at specific sites can impair the direct binding of transcription factors to their targets and, in turn, may lead to transcriptional downregulation (5–8), these epigenetic signals are also interpreted by specific proteins (9). These proteins have been classified into three families (10–12) according to their methyl-DNA binding domain: the methyl-CpG binding domain (MBD) proteins; the UHRF proteins that bind methylated DNA through there SRA domain proteins; and a subclass of zinc finger proteins that preferentially bind methylated DNA sequences (ZBTB33, ZBTB4, ZBTB38, ZFP57, KLF4).

MeCP2, MBD1, MBD2 and MBD4 are members of the MBD protein family that recognize methylated CpG sites independently of their surrounding sequences *in vitro* (13). In human cells and *Xenopus* oocytes these proteins are found associated with chromatin remodeling complexes along with histone deacetylases and/or histone methylases (14–18). The ability of these proteins to recruit repressor complexes at methylated CpG sites has suggested a direct relationship between DNA methylation and the establishment of a repressive chromatin architecture. However, more recent findings suggesting that MBD proteins may also be involved in other mechanisms such as alternative splicing and gene activation (19–21) have tempered this concept.

Several genome maps of MBD2 deposition have been constructed from human and mouse cells. Analysis of MBD2 binding sites at 25 000 promoter regions indicates that the promoter regions targeted by the endogenous MBD2 proteins are methylated and depleted for RNA polymerase II (22). Furthermore, parallel sequencing of chromatin immunoprecipitated fragments (ChIPseq) obtained from human HeLa and MCF7 cells expressing tagged-MBD2 vectors has shown that that MBD2 binding sites are methylated and that MBD2 deposition at TSS regions is associated with genes exhibiting repressive histone marks (21,23). A linear relationship between DNA methylation and MBD2 deposition is observed in mouse ES cells and derived neuronal cells expressing biotin-tagged MBD2 proteins from a single copy transgene (24). Although these studies show that a small fraction of MBD2 binding sites at promoter regions may be unmethylated and correspond to actively transcribed genes, these genome-wide analyses indicate that the presence of MBD2 at TSS regions is predominantly associated with methylated genes exhibiting a low transcriptional activity. Altogether, this suggests that MBD2 acts mainly as a methylation-dependent transcriptional repressor.

As expected from a transcriptional repressor involved in epigenetic mechanisms, MBD2 seems to play a role in the acquisition of specific phenotypes. MBD2 can block full reprogramming of somatic to iPS cells through direct binding to *NANOG* promoter elements thereby preventing transcriptional activation (25). In mice, MBD2 deletion alters the immune response (26), protects mice from hind-limb ischemia (27) and greatly reduces the number of intestinal adenoma in tumor-prone *APC*^*min*^ mice (28,29), mimicking the effects of experimentally induced DNA hypomethylation (30,31). Detailed gene candidate analysis indicates that MBD2 controls the expression of some exocrine pancreatic genes in a tissue-specific manner in mice (32). For example, *TFF2* is expressed in duodenum and silenced in colon, while this gene is methylated in both tissues. This tissue-specific repression is correlated with the tissue-specific presence of MBD2 at *TFF2* promoter and MBD2 deletion leads to *TFF2* upregulation in colon (32), suggesting that the dynamics of MBD2 binding has a direct effect on gene transcription. Taken together these data suggest that the cell-specific transcriptional repression occurring during differentiation or transformation may be associated, at least for some genes, with a redistribution of MBD2 proteins among methylated DNA regions.

In order to address this question we analyzed MBD2 binding sites in an isogenic cellular model of oncogenic transformation of human mammary epithelial cells constructed from the progression model described by Weinberg and colleagues (33). Human mammary cells were immortalized through the introduction of the human telomerase catalytic subunit gene (hTERT). The resulting immortalized cell line (HMEC-hTERT) was transformed by the introduction of the SV40 *T/t* antigens and oncogenic *H-RAS^V12^* genes (HMLER cell line). In a similar model constructed from human fetal lung fibroblasts (MRC-5 cells), it has been shown that DNA methylation changes occur predominantly during hTERT-induced immortalization while only subtle changes are associated with the transformation by SV40 large *T*-antigen and *H-RAS^V12^* (34). Thus, the comparison of HMEC-hTERT and HMLER cells offers the opportunity to investigate the potential modifications of MBD2 binding site associated with the acquisition of a specific phenotype in an isogenic background with a minor contribution of DNA methylation changes.

## MATERIALS AND METHODS

### Cell lines

HMEC-hTERT, HMLER, HME-ZEB1-RAS and mesenchymal HME-shP53-RAS cell lines were kindly provided by Anne-Pierre Morel (CRCL, Lyon, France) and cultured as previously described (35,36).

### Treatments

Cells were seeded at 1.10^5^ per well in six-well plates. The day after plating, cells were either transfected with 100 pmol of MBD2 targeting siRNA (siMBD2; sens: 5′-GGAGGAAGUGAUCCGAAAdTdT-3′) or control siRNA (siCtrl; Sigma-Aldrich, Saint Louis, MO, USA, MISSION siRNA Universal Negative Controls #1) using Lipofectamine-2000 (Sigma-Aldrich) as specified by the manufacturer's instructions, or treated every day with 10 μM of DAC (Sigma-Aldrich). Cells were collected 72 h after the start of the treatments.

### Western-blot

Around 1.0 × 10^6^ cells were lysed in NuPage LDS Sample Buffer and Reducing Agent (Life Technologies, Carlsbad, CA, USA). Lysates were sonicated and heated at 95°C for 10 min. After migration and transfer, membranes were incubated with either anti-MBD2 (Santa Cruz, Heidelberg, Germany, sc-9397) or anti-ß-Actin (Sigma-Aldrich, A5441) antibodies.

### RNA extraction

Total RNA was extracted using the NucleoSpin RNA kit (Macherey-Nagel, Hoerdt, France) following manufacturer's instructions. RNA purity, integrity and quantification were assessed using agarose gel-electrophoresis and analysis on a NanoDrop 1000 (Thermo Scientific, Wilmington, DE, USA). Pools of three to five independent extractions were sent for high-throughput sequencing to MGX-Montpellier GenomiX (library preparation with the TruSeq RNA sample preparation Kit from Illumina, followed by single-end 50 bp sequencing on Illumina HiSeq 2000).

### Digestion of genomic DNA

Genomic DNA was extracted from cell lines using the QIAamp DNA Mini Kit (Qiagen, Courtaboeuf, France). Two hundred micrograms of DNA were digested overnight at 37°C with either HpaII or MspI restriction enzymes (New England BioLabs, Evry, France), and analyzed on ethidium bromide-containing agarose gels.

### Methylated-DNA precipitation sequencing (MeDPseq)

Genomic DNA was sheared by sonication (final fragments ranged between 300 and 500 bp). Methylated-DNA precipitations (MeDP) were performed from 1 μg of sheared DNA using the MethylMiner Methylated DNA Enrichment Kit (Life Technologies). Library preparation and high-throughput sequencing (single-end 50 bp sequencing on Illumina HiSeq 2000) were performed at Beijing Genomics Institute (Hong-Kong, China) from pools of five independent experiments.

### Chromatin immunoprecipitation

MBD2 Chromatin immunoprecipitations (ChIP) were performed as previously described (37). Briefly, sheared chromatin (with a mean fragment length between 300 and 500 bp) was obtained by sonication of formaldehyde cross-linked nuclei. ChIP were then performed with a custom-made rabbit polyclonal antiserum obtained after immunization with peptides corresponding to the N-terminal part of the MBD2 protein (Covalab, Villeurbanne, Lyon) (38) using the ChIP Assay Kit (Merck Millipore, Saint-Quentin-en-Yvelines, France) as specified by the manufacturer's instruction. Precipitated DNA was finally purified using NucleoSpin Gel and PCR Clean-up kit, (Macherey-Nagel) according to the manufacturer's protocol ‘DNA clean-up of samples containing SDS.’

Input and bound fractions of DNA were dosed by fluorometry (Qubit 2.0, Life Technologies). Pools of five independent experiments were sent for high-throughput sequencing to ProfileXpert (Lyon, France) (library preparation and single-end 50 bp sequencing on Illumina HiSeq 2000).

Alternatively, enrichment in the bound fraction as compared to the input was measured by qPCR for several regions, using iQ SYBR Green supermix (BiroRad). Primer sequences and corresponding hybridization temperatures are listed in Supplementary Table S1.

### ChIP-chip and MeDP-chip

ChIP-chip and MeDP-chip experiments were performed as previously described (22). Briefly, chromatin fragments were precipitated with anti-MBD2 antibody (Covalab), and DNA fragments were precipitated using a His_6_-tagged recombinant protein containing four MBD domains cloned from MBD1 (39). The DNA fragments from the ChIP and MeDP experiments were amplified by random PCR. The samples were then labeled with the GeneChip WT Double-Stranded DNA Terminal Labeling Kit and hybridized to the human tiling arrays (Human Promoter 1.0R Arrays), which were then washed and scanned by ProfileXpert service (Lyon, France) according to Affymetrix protocols. Microarray signals were analyzed using aroma.affymetrix package on the hg18 reference genome.

### DNA methylation analysis

Bisulfite sequencing, used to determine the CpG methylation patterns of tens regions (Supplementary Table S2), was performed as described in (40). Briefly, after a first amplification using sequence-specific primers, PCR fragments were tagged in a second amplification step and sequenced using the Roche/454 GS junior system according to the manufacturer's protocol (Roche emPCR Amplification Method Manual – Lib-A and Roche Sequencing Method Manual). The mean number of reads per sample was 475, see Supplementary Table S2. Data were analyzed using Amplikyzer (https://pypi.python.org/pypi/amplikyzer/0.97).

### RNAseq analysis

Reads were aligned on the UCSC *Homo sapiens* hg19 genome using TopHat2 (41). Differential expression analysis was performed as described in (42), using the Bioconductor package edgeR (43). Only genes with at least 1 read per million (RPM) in at least two of the samples were kept for subsequent analyses. Enriched Gene Ontology terms and KEGG pathway were identified using Gene Set Enrichment Analysis (44) with genes pre-ranked according to their fold change induced by siMBD2 treatment.

### ChIPseq analysis

Reads were aligned on the UCSC *Homo sapiens* hg19 genome using Bowtie (45). Duplicate reads were filtered using SAMTools (46) to limit PCR induced biases. Preliminary peak detection was performed using Model-based Analysis of ChIP-Seq (MACS) (47). Over sequenced regions were defined as 500 bp regions containing more than three times the median number of reads in at least one of the two inputs. Peaks were filtered against these over sequenced regions, as they represent suspicious false positive regions. Peaks in HMEC-hTERT and HMLER were compared using MAnorm (48), and peaks common to both cell lines or cell line specific peaks (*P* value ≤ 0.01) were determined. Read density visualization at specific locations were drawn with Sushi.R (49). K-mean clusterization of peaks and subsequent visualization were performed with seqMINER (50). Integrative analysis of ChIPseq, RNAseq and ChIP-chip data was performed using the Bioconductor package Repitools (51). Top enriched motifs in peaks were obtained using the RSAT oligo-diff tool (52).

### Data deposition

The ChIPseq, the RNAseq and the microarray data from this publication have been submitted to the GEO database as entry GSE63237.

## RESULTS

### Identification of MDB2 binding sites and methylated DNA regions in HMEC-hTERT and HMLER cells

**Figure 1.**
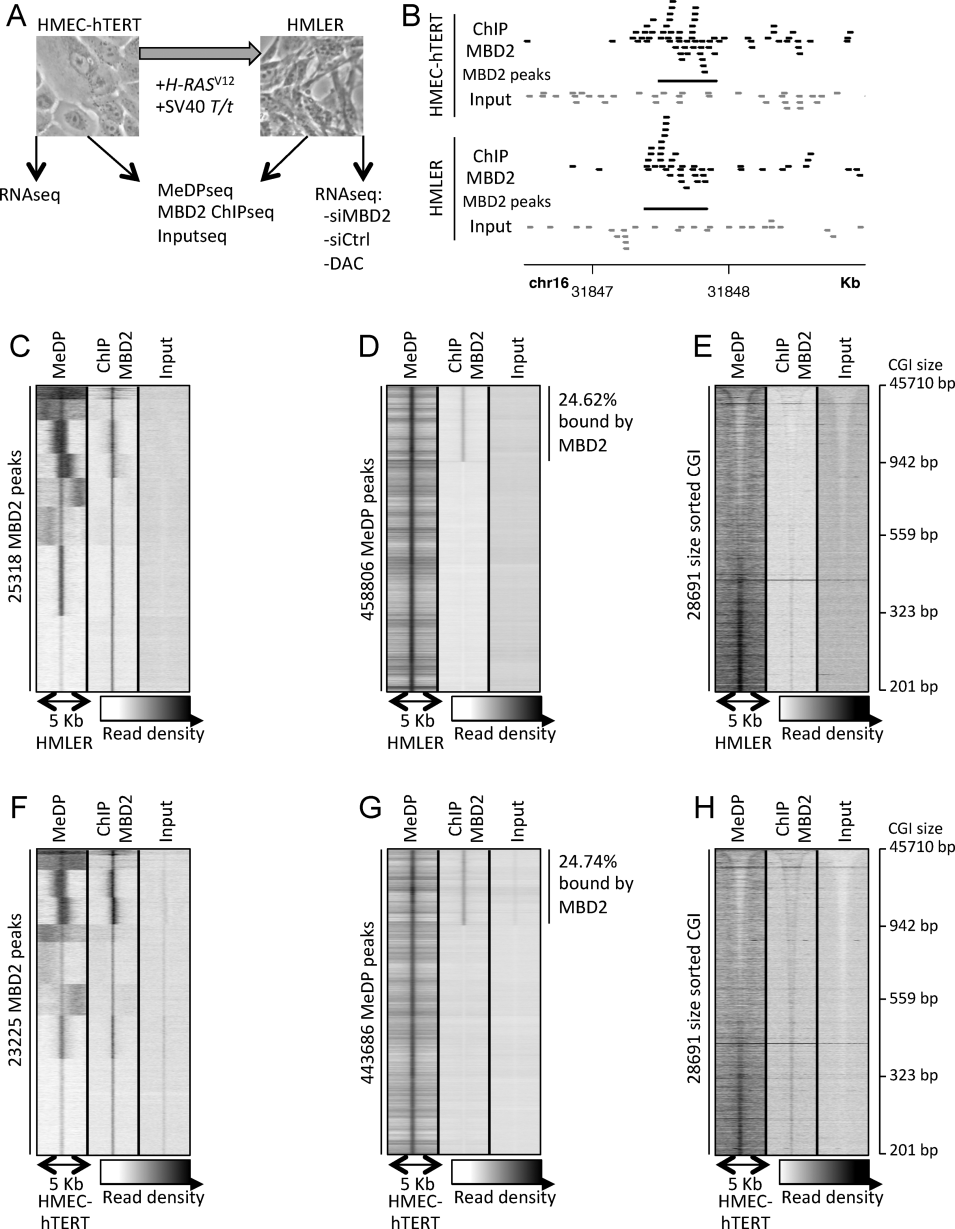
Endogenous MBD2 proteins bind methylated DNA regions. (**A**) Experimental scheme; the transformed mesenchymal HMLER cell line is derived from the immortalized epithelial HMEC-hTERT cells by oncogenic transformation using *H-RAS*^V12^ and SV40 *T/t* oncogenes. Sequencings were performed on a pool of five independent experiments. (**B**) Reads visualization at a MBD2 peak present in each cell line; MBD2 reads in black, input reads in gray, MBD2 peaks in black bars. Reads matching the + and − strands are on top and bottom, respectively. (**C** and **F**) Read density of MeDPseq, MBD2 ChIPseq and Input at each MBD2 peak. (**D** and **G**) Read density of MeDPseq, MBD2 ChIPseq and input at each MeDP peak. (**E** and **H**) Read density of MeDPseq, MBD2 ChIPseq and input at each CpG island (CGI), sorted by their size. (C to E) HMLER cell line. (F to H) HMEC-hTERT cell line.

To identify possible modifications of MBD2 deposition profiles associated with phenotype changes, genome-wide analyses (ChIPseq) were performed in an isogenic cellular model (Figure 1A) constructed from immortalized HMEC cells (HMEC-hTERT cells) transformed by expressing SV40 *T/t* antigen and *H-RAS^V12^* (33,35). In this study, we focused on the endogenous protein in order to take into account the potential changes in the regulation of MBD2 during the transformation process. In parallel, we identified methylated DNA regions by MeDPseq experiments performed in both cell lines using the MethylMiner kit (Invitrogen) for selecting methylated DNA regions.

**Figure 2.**
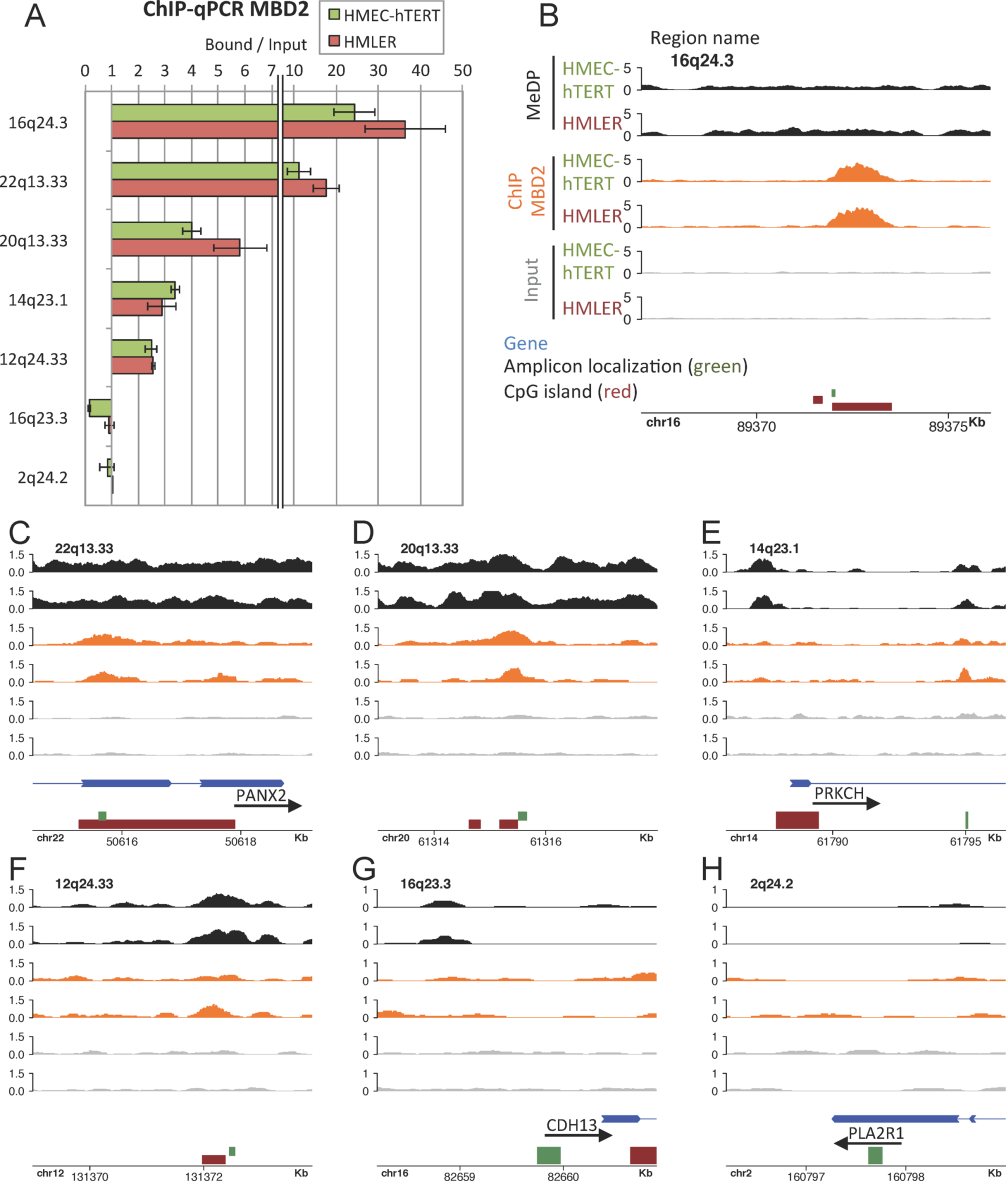
Concordance between ChIP-qPCR and ChIP-MBD2 at various genomic locations. (**A**) ChIP-qPCR experiments mapping MBD2 binding sites, in HMEC-hTERT cells (green) and HMLER cells (red). Each experiment was performed in duplicate. (**B**–**H**) Genome coverage for MeDPseq (black), MBD2 ChIPseq (orange) and Input-seq (gray) in HMEC-hTERT (upper tracks) and HMLER (bottom tracks) at the seven genomic location tested in A. Scales are in Fragments Per Million (FPM); Genes in blue, CpG islands in red; qPCR amplicon localization in green.

Methylated DNA regions and MBD2 peaks were identified from the aligned reads (sequenced fragments) (Figure 1B). These data were compared with ChIP-chip and MeDP-chip data obtained using an array representing 25,500 promoter regions (Human Promoter 1.0R array, Affymetrix). Methylated regions identified by MeDPseq were enriched in MeDP-chip experiments performed with recombinant proteins containing four MBD domains from MBD1 (22,39) (Supplementary Figure S1A and B). Similarly, MBD2 peaks identified from ChIPseq were enriched in MBD2 ChIP-chip (Supplementary Figure S1C and D). Next, a group of genomic regions sharing the same characteristics (MBD2 binding and DNA methylation) in both cell lines was analyzed by ChIP-qPCR (Figure 2A). Data obtained indicated a good concordance between the two methods, qPCR (Figure 2A) and determination of the distribution of MBD2 reads by ChIPseq (Figure 2B–H). We also validated 14 cell-specific MBD2 binding sites (Supplementary Figure S2). For many of these, DNA methylation changes did not seem to be associated with the gain of MBD2 binding sites in HMLER cells (Supplementary Figure S2 regions 14q11.2, 12q11.22, 8q22.2 and 16p13.3). Ten of these regions were amplified by PCR from bisulfite-modified DNAs. Parallel sequencing of the PCR fragments validated their methylation status deduced from MeDP-seq experiments (Supplementary Table S2). On all the regions analyzed by qPCR, we confirmed that depletion of MBD2 by siRNA strongly reduced ChIP efficiency (Supplementary Figure S3), confirming the specificity of the antibody.

### Endogenous MBD2 binding sites are methylated

MBD2 binding sites (2.5 kb up and down of the center of each peak) were visualized by heat maps showing read density from the MBD2 ChIPseq (MBD2-reads), the MeDPseq (MeDP-reads) and the negative control, Input-seq. As expected, strong signal intensities were observed when MBD2-reads were plotted against MBD2 peaks (Figure 1C, HMLER cells and 1F, HMEC-hTERT cells). The centers of MBD2 peaks were also enriched in MeDP-reads (Figure 1C and F), indicating that high density in DNA methylation signals was associated with MBD2 binding sites. However, K means clustering showed that a subset of MBD2 peaks is associated with weakly or unmethylated DNA regions (Figure 1C and F). Conversely, DNA methylation peaks identified by MeDPseq were enriched in MeDP-reads (Figure 1D and G).

The clustering of MeDP positive regions according to their MBD2 ChIPseq read densities suggested that up to a quarter of methylated DNA regions were bound by endogenous MBD2 (Figure 1D and G). Altogether, these data indicated that, in HMEC-hTERT and HMLER cells, the endogenous MBD2 proteins associated predominantly methylated DNA regions, as previously reported from ectopic expression of tagged-MBD2 proteins in other cellular models (23,24,53). The relationship between DNA methylation and MBD2 binding was also observed at Alu sequences. Alu sequenced are classified into three main groups depending on the age of the sequence (RepeatMasker table download from UCSC). The younger sequences, AluY, were found to be more methylated than the AluJ (older Alu sequences). The AluS, intermediate in age, exhibited an intermediate level of DNA methylation (Supplementary Figure S4C). The distribution of MBD2 read densities on these Alu sequences was parallel to the DNA methylation profiles (Supplementary Figure S4B).

When CpG islands (CGIs, UCSC database) were classified according to their length and plotted against MeDP-reads, the shorter CGIs tended to exhibit an increased read density (Figure 1E and H). These short CGIs were also enriched in MBD2 reads (Figure 1E and H), showing that MBD2 proteins targeted CpG-rich sequences when methylated. The clustering of CGIs according to their MBD2 ChIPseq or MeDPseq read densities led to the identification of a CGI subgroup where MBD2 binding or DNA methylation were detectable mainly on the shore of the islands, while another subgroup shown a preferential enrichment at the core sequences (Supplementary Figure S5A and D).

Identification of enriched K-mer in MBD2 peaks using RSAT tool failed to identify any sequence specificity except the presence of CpG sites (Supplementary Table S3). Altogether, these data indicated that DNA methylation is the main parameter driving MBD2 deposition.

### MBD2 is a major DNA methylation-dependent transcriptional repressor

Transcriptomes of HMEC-hTERT and HMLER cells were determined from RNAseq experiments. Genes with a methylated DNA region within ±1 kb of their TSS showed a low transcriptional level (Supplementary Figure S5). Similarly, the transcriptional level of genes with MBD2 peaks within ±1 kb of their TSS was low (Supplementary Figure S6). Thus MBD2 binding of promoter was linked to gene silencing.

**Figure 3.**
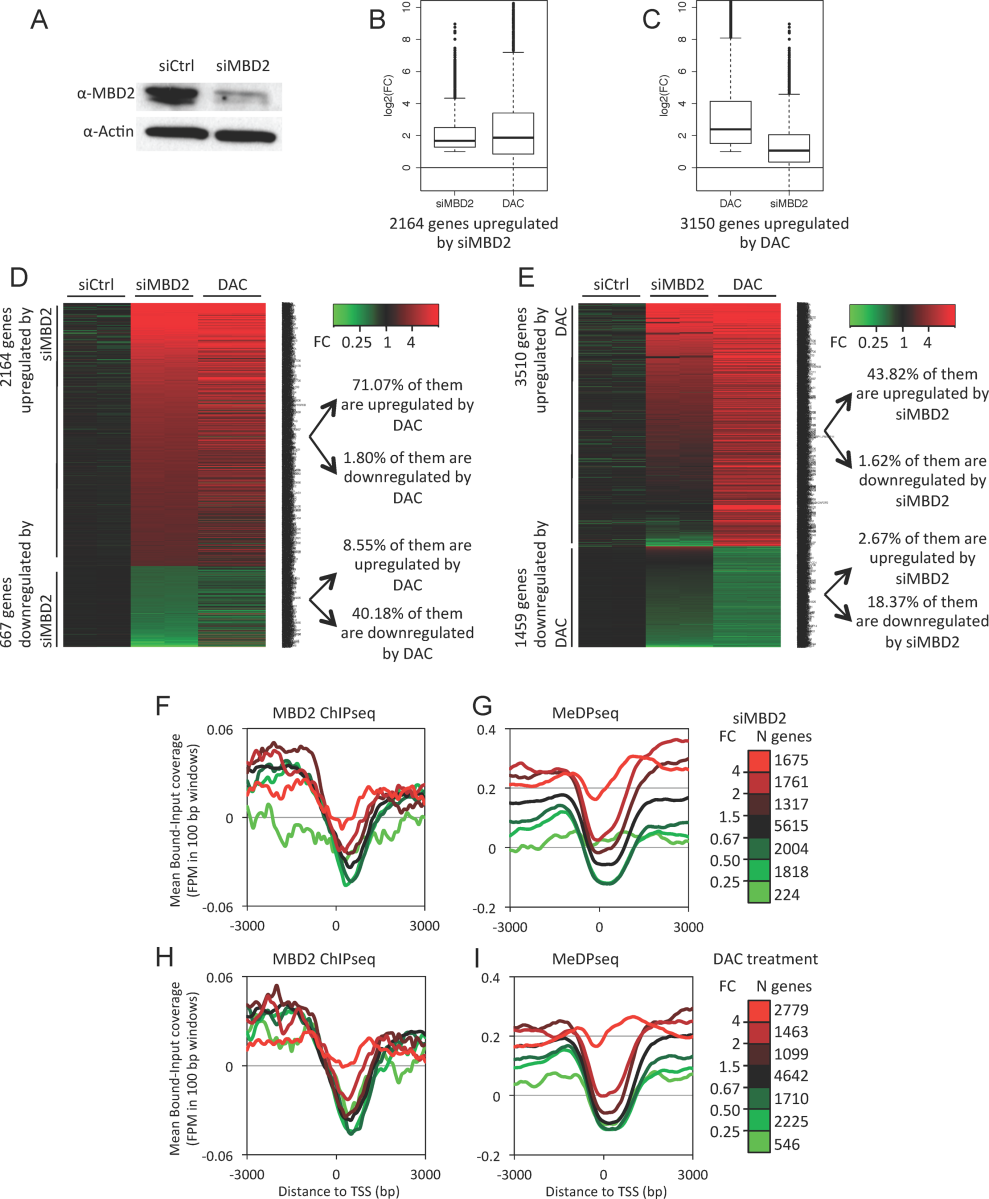
MBD2 is a methylation-dependent transcriptional repressor in HMLER cells. (**A**) Immunoblot analysis of MBD2 proteins in siRNA treated HMLER cells. (**B**) Box plots depicting the fold changes (FC) of the transcription levels of genes upregulated upon MBD2 depletion (siMBD2) and the FC of the same genes in 5-aza-deoxycytidine treated cells (DAC). The upregulated genes were defined as genes exhibiting a FC ≥ 2 with a *P* value ≤ 0.01. (**C**) Box plots depicting the FC of the transcription levels of genes upregulated upon DAC treatments and the FC of the same genes in MBD2-depleted HMLER cells. (**D**) Heat maps of genes upregulated or downregulated upon siRNA targeting MBD2. Most genes upregulated by MBD2 depletion were also upregulated by DAC treatments. (**E**) Heat maps of genes upregulated or downregulated upon DAC treatments. About 44% of the genes upregulated upon DAC treatments were also upregulated by MBD2 depletion. (**F** to **I**) Integrative analysis of ChIPseq and RNAseq. Mean coverages at gene promoters were plotted according to their responses to MBD2 siRNA or DAC treatments. Genes were classified into seven groups according to their responses to either MBD2 depletion or DNA methylation inhibition. N genes: number of genes in each group. For each gene cluster, mean MBD2-read densities and mean MeDP-read densities at the TSS (± 3 kb) were determined. (F) MBD2-read density at TSS regions of genes classified by their response to siMBD2. (G) MeDP-read density at TSS regions of genes classified by their response to siMBD2. (H) MBD2-read density at TSS regions of genes classified by their response to DAC treatments. (I) MeDP-read density at TSS regions of genes classified by their response to DAC treatments.

The effects of MBD2 depletion and DNA methylation inhibition on gene transcription were then assessed. High-throughput sequencing of poly-adenylated RNA (RNAseq) were performed with RNA extracted from HMLER cells treated with either siMBD2 (siRNA targeting *MBD2* transcripts (37,54)) or 5-aza-deoxycytidine (DAC). Non-specific siRNA was used as a control. The analysis of the data was performed from duplicate RNAseq experiments (Supplementary Table S4). RNAseq analysis indicated a reduction of 89.5 ± 0.33% of MBD2 mRNA in siMBD2-treated HMLER cells, while the levels of MeCP2 and MBD1 transcripts were not affected (Supplementary Table S4). This downregulation was also observed at protein level as shown by western blot analysis (Figure 3A). Digestion of genomic DNA with HpaII, a restriction enzyme inhibited by CpG methylation, indicated that DAC induced a global DNA hypomethylation of HMLER cells (Supplementary Figure S7). Parallel sequencing of PCR fragments indicated that all the nine methylated genes analyzed were hypomethylated after DAC treatments (Supplementary Table S2). The classification of the sequenced molecules according to their methylation patterns indicated that this hypomethylation was not randomly distributed but corresponded to a mixture of fully demethylated molecules and unaffected molecules (data not shown).

Among the 14,814 genes whose expression level could be reliably assessed in our experiment (see materials and methods section), 14.6% (2,164) were upregulated (fold change greater than 2, and *P*-value ≤ 0.01) upon siMBD2 treatments. In line with the previous experiments showing that MBD2 associated methylated DNA regions (Figure 1), a large proportion (∼70%) of these upregulated genes were also found upregulated in HMLER cells treated with DAC (Figure 3B and D). Conversely, a subset of genes (∼40%) upregulated in DAC-treated HMLER cells was also upregulated upon MBD2 depletion (Figure 3C and E).

Cross analyses of RNAseq, MBD2 ChIPseq and MeDPseq experiments were performed by plotting MBD2-read densities and MeDP-read densities at the TSS (±3 kb) of genes classified in seven clusters according to their responses to either MBD2 depletion or DNA methylation inhibition. These data are summarized in Figure 3F–I and indicated that the upregulated genes upon MBD2 depletion were enriched in MBD2-reads near their TSS in untreated cells (Figure 3F, red curves). Therefore, MBD2 gene repressions were likely due to MBD2 binding at promoter regions. As expected from the correlation between MBD2 binding and DNA methylation, the TSS regions of these upregulated genes were also enriched in MeDP-reads. (Figure 3G, red curve). Conversely, the genes upregulated upon hypomethylation of HMLER cells were enriched in MBD2-reads and MeDP-reads (Figure 3H and I, red curves).

Enhancers are distal regulatory elements that can impact gene transcription levels. Andersson *et al*. (55) identified 66,942 enhancer-TSS association in the human genome. The clustering of these enhancers according to their MBD2 ChIPseq (Supplementary Figure S8A) or MeDPseq read densities (Supplementary Figure S8B) indicated that 6.412 and 10,896 enhancers were bound by MBD2 or methylated, respectively, in HMLER cells. Genes associated with MBD2-positive enhancers or methylated enhancers were significantly underexpressed (Supplementary Figure S8C). A subset of these genes was upregulated upon treatments with siMBD2 (Supplementary Figure S8D) or with DAC (Supplementary Figure S8E).

Genes close to a CGi, exhibiting a methylated or a MBD2 positive core region, were expressed at a low level when compared with genes associated with unmethylated/MBD2-free CGi (Supplementary Figure S5B and E). Furthermore, DAC and siMBD2 treatments led to an upregualtion of the genes associated with methylated/MBD2-positive CGi-core regions (Supplementary Figure S5C and F). Although significantly less affected, the transcription level of genes associated with methylated or MBD2-positive CGi-shore regions were also downregulated (Supplementary Figure S5B and E), while inhibition of DNA methylation or MBD2 depletion upregulated these genes (Supplementary Figure S5C and F). Thus, the repressive forces of DNA methylation and MBD2 binding seem to be positively correlated with CpG density.

A similar effect of MBD2 depletion or inhibition of DNA methylation was also observed at repeated elements. Depletion of MBD2 or DAC treatments induced a small increase of the steady state level of transcripts corresponding to AluY elements while the transcription level of the unmethylated and MBD2-free AluJ elements were unaffected by these treatments (Supplementary Figure S4A).

These data highlighted the role of MBD2 in the DNA methylation-dependent gene repression and indicated that, for a large subset of genes, a loss of MBD2 or a demethylation have similar effects leading to their upregulation. Thus, we investigated the contribution of these two epigenetics events, MBD2 deposition and DNA methylation, on the downregulation associated with the transformation of immortalized HMEC-hTERT cells.

### Dynamics of MBD2 deposition during oncogenic transformation

**Figure 4.**
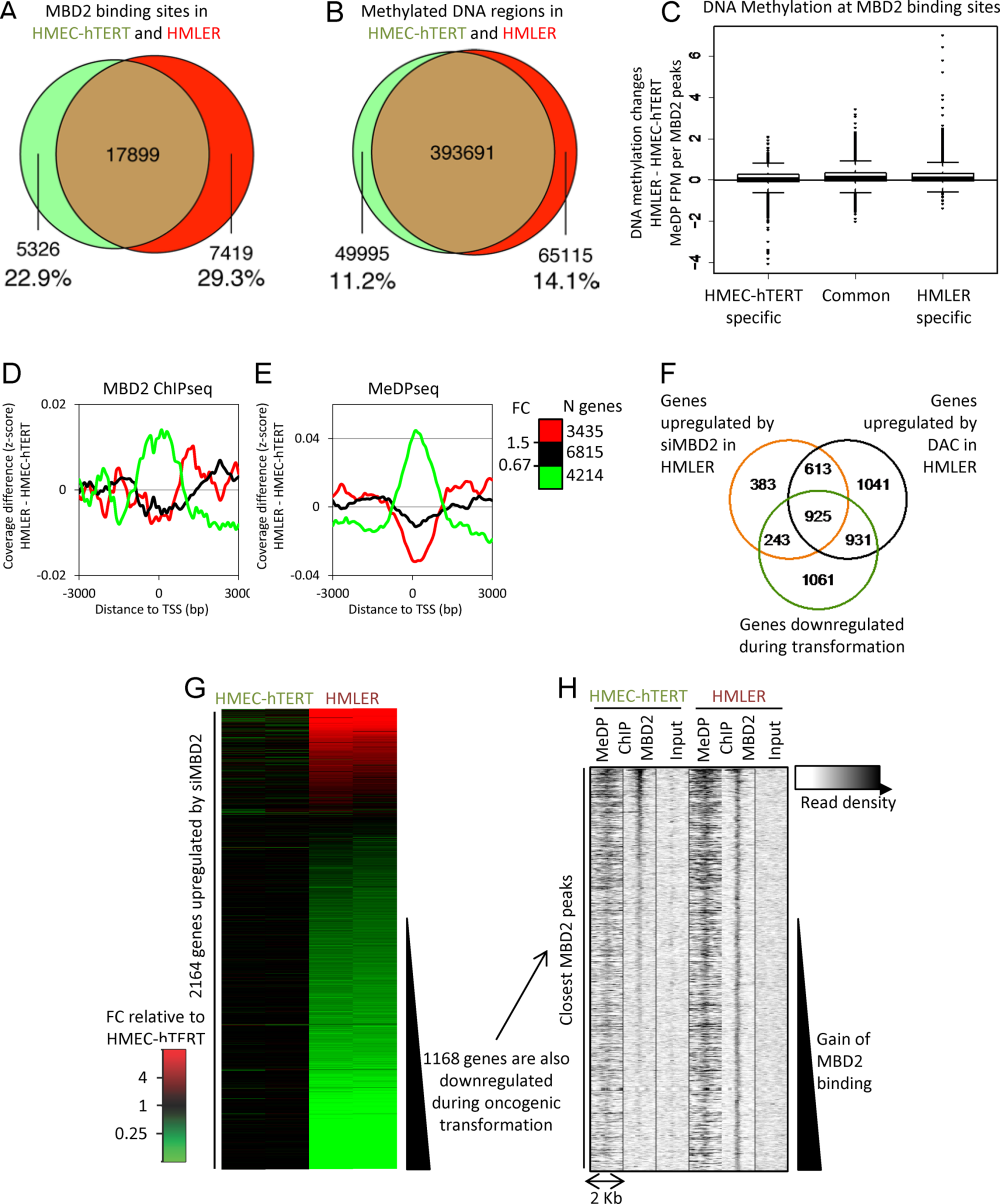
Dynamics of MBD2 distribution during oncogenic transformation. (**A**) Venn diagram depicting the overlap between MBD2 binding sites of HMEC-HTERT and HMLER cells; in green: regions that significantly lose MBD2 during transformation (*P* value ≤ 0.01), in red: regions that significantly gain MBD2 during transformation (*P* value ≤ 0.01), in brown: common regions. (**B**) Venn diagram depicting the overlap between methylated DNA regions of HMEC-HTERT and HMLER cells; in green: regions that significantly lose DNA methylation during transformation (*P* value ≤ 0.01), in red: regions that significantly gain DNA methylation during transformation (*P* value ≤ 0.01), in brown: common methylated DNA regions. (**C**) DNA methylation changes at MBD2 binding sites. Differences in MeDP coverage (FPM, Fragments Per Million) at MBD2 peaks were computed for regions bound by MBD2 in both cell lines, losing MBD2 or gaining MBD2 in HMLER when compared with HMEC-hTERT. (**D** and **E**) Genes were classified according to their differential expression level (FC) between HMEC-HTERT and HMLER cells. (D) Genes downregulated during oncogenic transformation gain MBD2-reads near their TSS (red curve), in contrast to unaffected (black curve) or downregulated genes (green curve). (E) Genes downregulated during oncogenic transformation exhibited gain of MeDP-reads near their TSS (red curve), in contrast to unaffected (black curve) or downregulated genes (green curve) (**F**) Venn diagram depicting the overlap between genes upregulated upon MBD2 depletion, DAC treatments and genes downregulated in HMLER cells when compared with HMEC-hTERT cells. A large proportion of genes downregulated during oncogenic transformation was also upregulated upon MBD2 depletion and DNA methylation inhibition in the transformed HMLER cell line. (**G**) Transcription level modification between HMEC-hTERT and HMLER, of the 2,164 genes upregulated after a siMBD2 treatment in HMLER. Around 1,168 of these genes are expressed at least two times less in HMLER than in HMEC-hTERT. (**H**) Read densities from MeDPseq, MBD2 ChIPseq and Input-seq in HMEC-hTERT and HMLER, at the closest MBD2 peaks in HMLER cells form the TSS of the 1,168 genes identified in (G). Around half of these regions shown an increase MBD2 ChIPseq read density in HMLER as compared to HMEC-hTERT.

Differential MBD2 binding profiles between HMEC-hTERT and HMLER cells were determined by quantitative comparison of MBD-peaks (48) identified by ChIPseq analyses. A high number (17,899) of MBD2 peaks was not affected by the transformation process (Figure 4A). However, 5,326 regions (22.9%) associated by MBD2 in HMEC-hTERT cells lost MBD2 in HMLER, while 7,419 (29.3%) regions gained MBD2 binding in HMLER cells when compared with MBD2 profiles in HMEC-hTERT (Figure 4A). DNA methylation changes were more limited in proportion as HMEC-hTERT and HMLER cells shared 393,631 DNA methylation peaks while 49,995 regions (11.2%) were HMEC-hTERT specific and 65,115 regions (14.1%) were HMLER specific (Figure 4B).

To investigate if MBD2 redistributions were due to local DNA methylation changes, we analyzed changes in MeDPseq read density at MBD2 peaks shared by both cell lines, or unique to either HMEC-hTERT or HMLER (Figure 4C). The mean values (number of HMLER MeDP-reads minus HMEC-hTERT MeDP-reads) were near zero for all MBD2-peak categories. However, a few outliers did show a strong decrease of DNA methylation in regions losing MBD2, and a strong increase of DNA methylation in regions gaining MBD2. ChIP-qPCR experiments have exemplified this latter point at several chromosomal locations such as 10q11.22, 10p15.1 and 1p36.33 (Supplementary Figure S2A).

Thus, at some methylated sites MBD2 deposition was not observed in HMEC-hTERTcells, while these sites were associated by MBD2 in HMLER cells. These data indicated that MBD2 deposition is a dynamic process not exclusively driven by DNA methylation changes.

### MBD2 is involved in the repression of genes downregulated in oncogenic HMLER cells

Interestingly, genes downregulated during the transformation of HMEC-hTERT gained MBD2-reads and MeDP-reads at TSS regions (Figure 4D and E, red curves). Furthermore, more than 50% (1,168 of 2,164) of genes upregulated upon MBD2 depletion in HMLER cells were genes downregulated during the transformation process (Figure 4F and G). Read densities at the MBD2 peaks nearest to the annotated TSS of these 1,168 genes were visualized by heat maps showing read density from the MBD2-reads and the MeDP-reads. At least half of these regions were enriched in MBD2 in HMLER cells when compared with HMEC-hTERT cells (Figure 4H).

**Figure 5.**
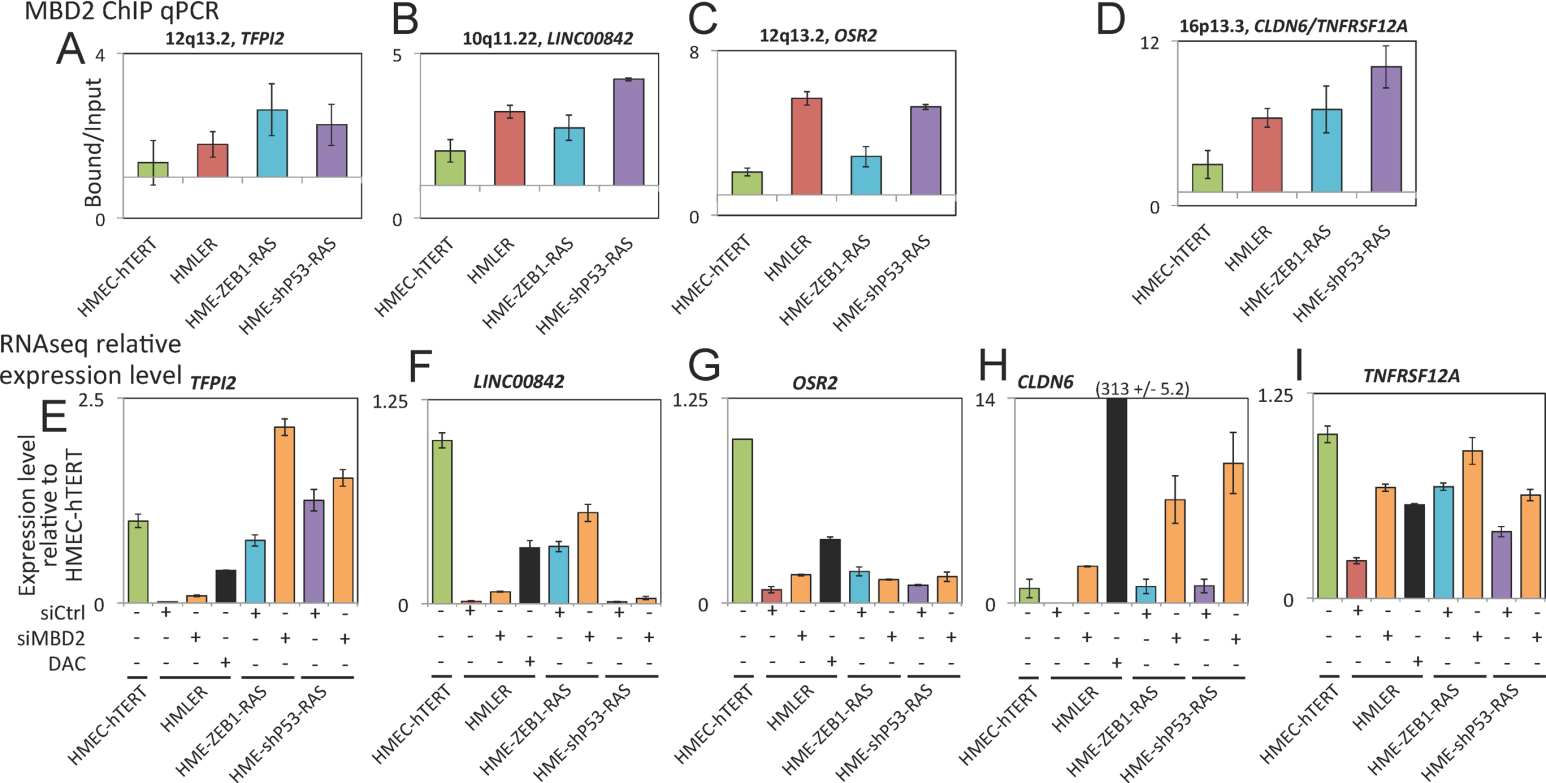
Examples of genes repressed by MBD2 during oncogenic transformation. (**A**–**D**) MBD2 ChIP-qPCR at four genomic regions corresponding to five gene promoters in the HMEC-hTERT cell line and the transformed HMLER, HME-ZEB1-RAS and HME-shP53-RAS cell lines. These regions exhibited an increase of MBD2 binding during oncogenic transformation. Each experiment was performed two times independently. (**E**–**I**) Relative expression of five genes in HMEC-hTERT, HMLER treated with a control siRNA (siCtrl), a siRNA targeting *MBD2* (siMBD2) or 5-aza-deoxycytidine (DAC), HME-ZEB1-RAS treated with a control siRNA or a siRNA targeting *MBD2* and in HME-shP53-RAS treated with a control siRNA or a siRNA targeting *MBD2*. Expression levels were estimated from RNA sequencing duplicates.

Data obtained for *TFPI2, LINC00842, OSR2, CLDN6* and *TNFRSF12A* genes illustrate this analysis (Figure 5E–I and Supplementary Figure S9A). MBD2 deposition near the TSS of these genes was increased during oncogenic transformation (Supplementary Figure S2 and Figure 5A–D). These genes were downregulated during oncogenic transformation. Depletion of MBD2 by siRNA led to an upregulation of these genes in the transformed cell line HMLER (Figure 5E-I). These data indicated that MBD2 participated in the establishment of transcriptomic changes induced by oncogenic transformation.

### Dynamics of MBD2 deposition upon oncogenic transformation of immortalized human mammary cells by ZEB1 expression or P53 depletion

**Figure 6.**
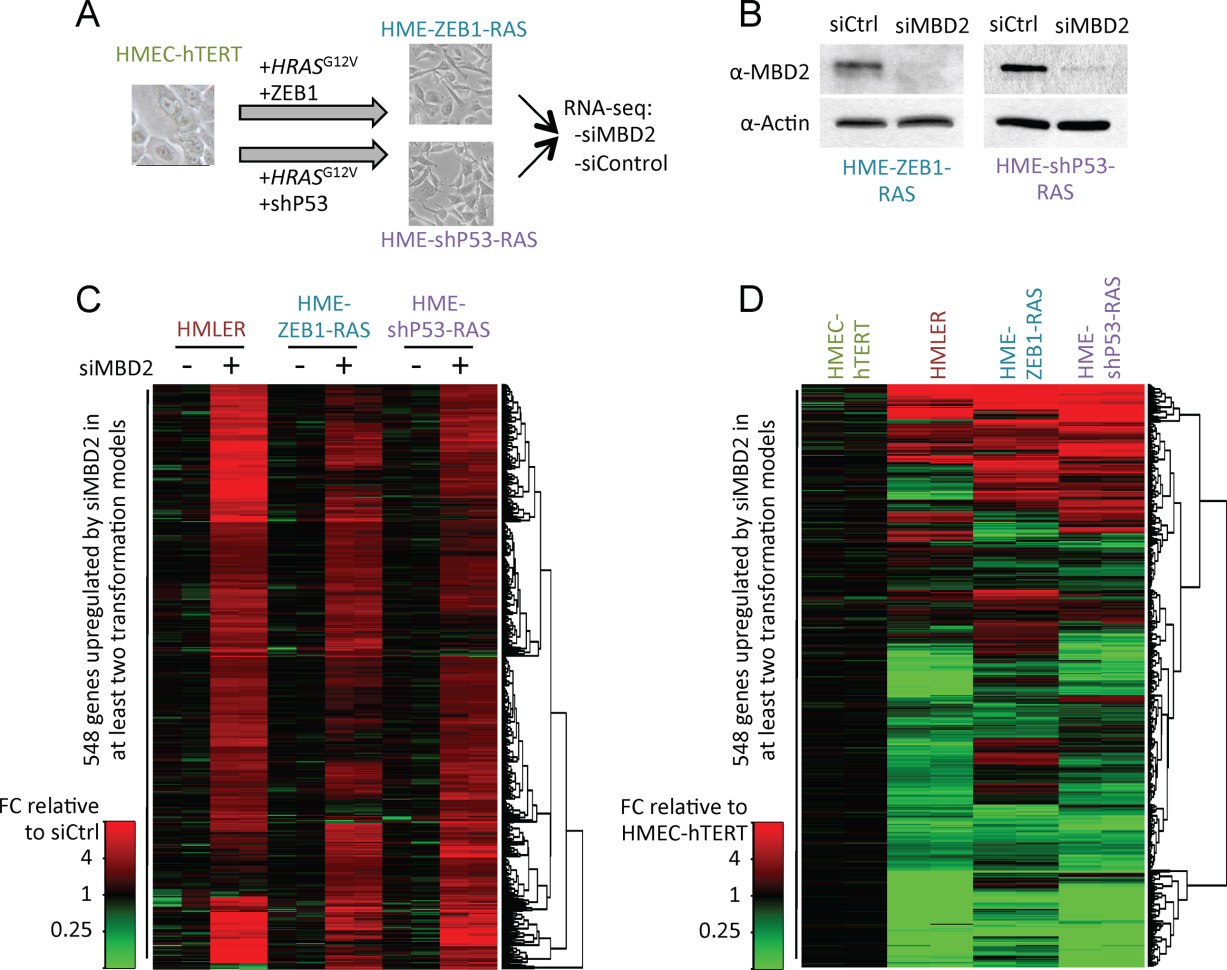
MBD2 repressed a common set of genes during oncogenic transformation. (**A**) Experimental scheme of generation of two cell lines from HMEC-hTERT cells by oncogenic transformation. HME-ZEB1-RAS cells were obtained by the expression of the embryonic transcription factor ZEB1 and H-RAS^V12^, and HME-shP53-RAS cells were obtained by the expression of a shRNA targeting P53 and H-RAS^V12^ genes. (**B**) Immunoblot analysis of MBD2 protein level in HME-ZEB1-RAS and HME-shP53-RAS upon siRNA treatment targeting *MBD2*. (**C** and **D**) Heat maps summarizing genes downregulated (green) or upregulated (red). (C) A set of 548 genes was upregulated in MBD2 knockdown cell lines. (D) Most of these genes upregulated by siRNA against MBD2 in transformed cell lines were genes that are downregulated during oncogenic transformation.

Transcriptomic analyses of HMLER cells treated with siMBD2 or siControl indicated that MBD2 depletion led to an upregulation of genes that are silenced upon the transformation of HMEC-hTERT cells by expressing SV40 *T/t* antigens and *H-RAS^V12^* genes. It has been shown that the expression of SV40-derived sequences affects several other pathways in addition to pRB and P53 (56), raising the possibility of a SV40-dependent redistribution of MBD2 proteins. Among the mechanisms able to cooperate with the expression of mutated *H-RAS* during neoplastic processes, the expression of the transcription factor *ZEB1* or the disruption of P53-pathways seem to play crucial roles (57,58). Thus, the involvement of MBD2 in the control of gene expression was investigated in two cellular models (Figure 6A) in which SV40 *T/t*-antigens expression were replaced by *ZEB1* gene expression (HME-ZEB1-RAS cell line) or a shRNA targeting *P53* transcripts (HME-shP53-RAS cell line) (35,36). When induced, both cell lines exhibited, as the HMLER cells, a mesenchymal phenotype and malignant properties (33,35,36).

We induced MBD2 depletion in HME-ZEB1-RAS and HME-shP53-RAS cells by siMBD2 treatments, and differential gene expression profiles between siMBD2 and siControl treated cells were determined from RNAseq experiments (Figure 6A). Transient transfection of siMBD2 resulted in a reduction of 89.0 ± 0.33% and 91.3 ± 0.25% of MBD2 transcripts in HME-ZEB1-RAS and HME-shP53-RAS cells, respectively (Supplementary Table S3), and a large decrease in MBD2 proteins was also observed from western blot analyses (Figure 5B).

**Figure 7.**
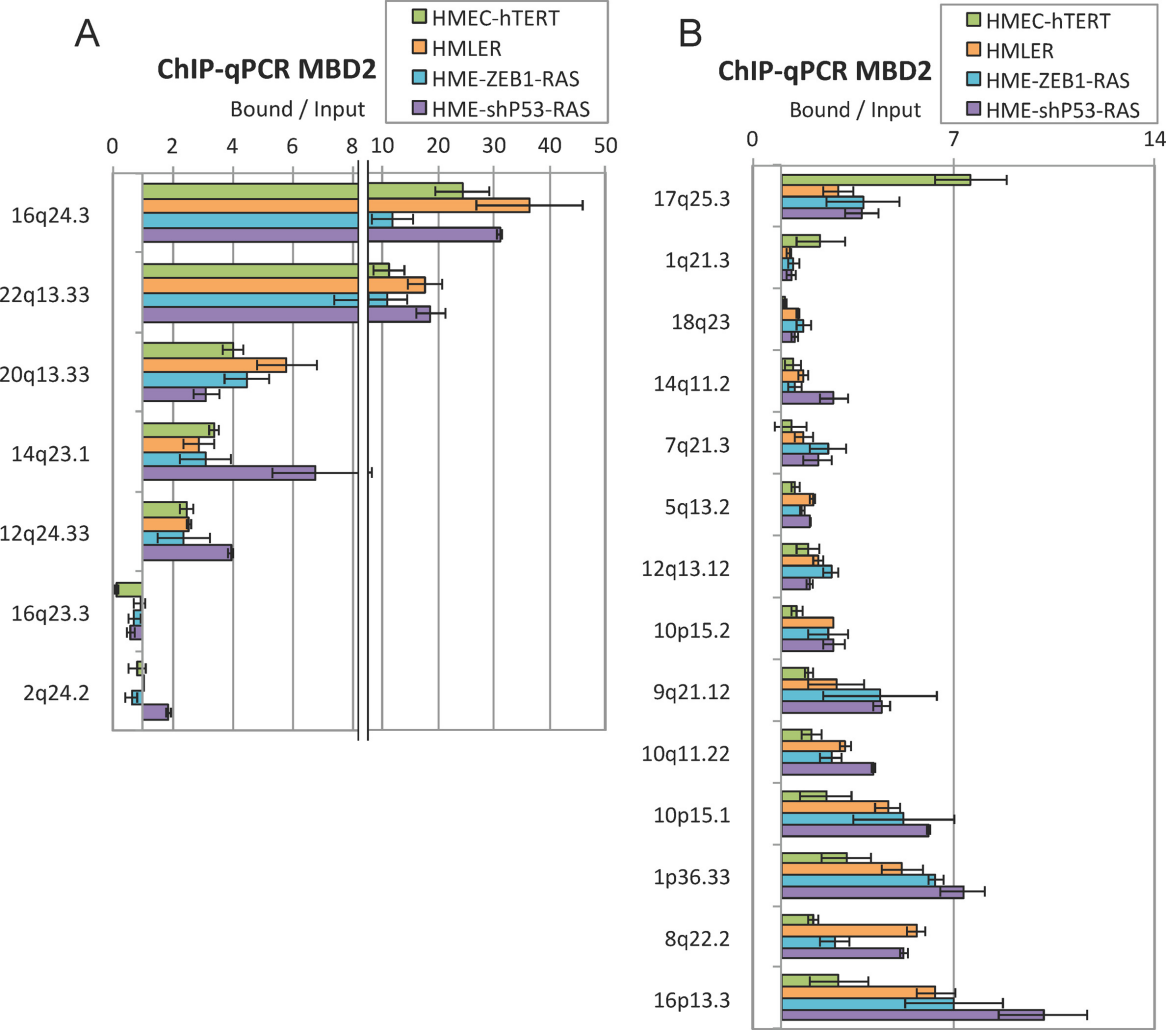
MBD2 ChIP-qPCR experiments from HMEC-HTERT, HMLER, HME-ZB1-RAS and HME-shP53 cell lines. Error bars represent average deviation from two independent experiments. (**A**) MBD2 binding at several positive and negative regions. (**B**) MBD2 redistribution in the genome during oncogenic transformation was frequently concordant in the different transformed cell lines.

We identified 584 genes upregulated by MBD2 depletion in at least two cell lines (Figure 5C, fold change ≥ 2, *P* value ≤ 0.01). A large subset (247, 42.3%) of these genes were downregulated during oncogenic transformation (Figure 6D). According to our ChIPseq data, at least half of the MBD2 peaks near the TSS of these 247 genes shown an increase MBD2 binding in HMLER as compare to HMEC-hTERT (Supplementary Figure S10). MBD2 ChIP-qPCR assays performed at genomic locations previously analyzed (Figure 2 and Supplementary Figure S2) indicated that MBD2 binding profiles identified in HMLER cells were frequently similar in HME-ZEB1-RAS and HME-shP53-RAS cells (Figure 7). Notably, various losses (17q25.3, 1q21.3) or gains (9q21.12, 10p15.1, 1p36.33, 8q22.2, 16p13.3) of MBD2 binding during oncogenic transformation were reproduced in these two models (Figures 5A–D, 7B and Supplementary Figure S9B). As expected from genome-wide data, parallel sequencing of PCR fragments obtained from bisulfite-modified DNA indicated that MBD2 deposition was correlated with DNA methylation in HME-ZEB1-RAS and HME-shP53-RAS cells at the 10 genes analyzed (Supplementary Table S2).

Altogether, these data suggest that MBD2 is involved in the regulation of gene expression during the transformation of HME-ZEB1-RAS and HME-shP53-RAS cells. This role is thus likely to be independent of the strategies involved in the abrogation of oncosuppressive barriers.

### Genes repressed by MBD2 in transformed cell lines may contribute to the epithelial phenotype

Genes upregulated by MBD2 depletion in HMLER, HME-ZEB1-RAS and HME-shP53-RAS were investigated for their Gene Ontology terms and their KEGG pathways contributions by Gene Set Enrichment Analysis (GSEA). Enriched Gene Ontology terms were of various natures but include EXTRACELLULAR_REGION, BASEMENT_MEMBRANE, INTEGRIN_COMPLEX (Supplementary Table S5). Enriched KEGG pathways included KEGG_CELL_ADHESION_MOLECULES_CAMS, KEGG_FOCAL_ADHESION (Supplementary Table S4). Although genes repressed by MBD2 in the transformed cell lines showed a wide diversity of functions, some of them appeared to be associated with the epithelial phenotype. Their downregulation by MBD2 may contribute to the epithelio-mesenchymal transition induced by the oncogenic transformation of these cells.

## DISCUSSION

It has long been known that DNA methylation patterns are altered in cancer cells (59,60), these modifications include global loss of 5-methylcytosines and local hypermethylations (61,62). Aberrant methylations at CpG islands have been extensively studied in past years since such hypermethylations have been found at the 5′ regulatory sequences of many tumor suppressor genes and associated with their silencing (3,63). Although the expression level of genes in their tissue of origin may influence their methylation status in the corresponding cancer cells (64), genome-wide studies have shown that hundreds of genes exhibiting hypermethylated CpG island at their 5′ end were never methylated from embryonic development onward (3).

From the first studies using *in vitro* methylated gene expression vectors, accumulating evidence indicates that the methylation of CpG rich sequences at the 5′ end of a gene provides a strong signal for maintaining/inducing gene silencing (65,66). In cell lines, inhibition of DNA methylation by cytosine analog (17) or DNMT1 knockdown (67,68) leads to the re-expression of many methylated genes. This relationship has been also observed in living animals. For example, conditional inactivation of DNMT1 causes aberrant gene expression of many genes in mouse fibroblasts (69).

Histone deacetylation, histone methylation and chromatin compaction are major events involved in gene repression and DNA methylation is associated with these chromatin modifications (70,71). The importance of proteins associating DNA sequences was suggested many years ago by *in vitro* transcription assays showing that the methylation of templates does not prevent transcription (72), while methylated vectors are not transcribed in living cell (65,73). From these pioneering works, it had been established that specific proteins from different families recognize methylated DNA. Among these proteins, the proteins containing a methyl-CpG binding domain were the first discovered (13,74) and many studies have indicated that the MBD proteins are good candidates for interpreting the methylation signals (9,10). In contrast with transcription factors, the sequence specificities of these proteins does not seem to be very stringent. *In vitro* assays indicate that MBD1 has a higher affinity for meCGCA or TGmeCGCAsites (75), while MeCP2 preferentially associates methylated CpG followed by a run of four or more A/T bases (76). However, the presence of symmetrically methylated CpGs seems to be the major determinant of their binding since multiple methylated CpGs can override their sequence preference at methylated sites (75,76). Biochemical studies of MBD2 have also indicated a preference for a short sequence CmeCGA (77). In line with this finding, CCG sequences are preferentially found in many MBD2 genomic targets (77). However, this observation might be biased by over-representation of methylated CpG-rich sequences among the genomic targets of MBD2 (37,78–82).

The intracellular concentrations of each MBD protein may also influence their targeting, for example, in neurons MeCP2 is highly expressed and tracks methyl CpG densities (83). In HeLa cells, clearance of MBD2 by siRNA at the BRCA1-NBR2 locus does not promote the binding of the endogenous MeCP2 or MBD1 proteins. However, overexpression of a vector coding for MeCP2 induces its binding at this site (37). In mice, knockdown of the individual MBD proteins, MeCP2, MBD1 or MBD2 (84–86) results in the upregulation of many genes but not in global misexpression of endogenous methylated genes. Thus, other members of the family might compensate for the absence of a specific MBD since many cell and tissue types express multiple MBD proteins. Nevertheless, the deletion of a specific MBD protein leads to specific phenotypes (10), indicating a functional specificity for these proteins and gene candidate analysis has provided examples of gene specific upregulation.

Altogether, these data argue against a full redundancy between MeCP2, MBD1 and MBD2, and suggest that the distribution of MBD proteins across the methylated regions might represent an additional level in the interpretation of the DNA methylation marks. Indeed, our analysis of the MBD2 binding profiles during the oncogenic transformation of immortalized human mammary cells indicates that the modifications of MBD2 distribution are, for a subset of genes, independent of DNA methylations changes.

Although chromatin immuno-precipitation of endogenous MBD proteins has proven challenging (23,24,53), we have performed ChIPseq against endogenous MBD2 proteins in two isogenic cell lines in order to take into account the cell-specific regulation of MBD2. Despite the significant differences in technologies, ChIPseq data correlated well with those obtained from MBD2 ChIP-chip experiments. ChIP-qPCR on arbitrary chosen negative or positive regions also showed a good concordance between data obtained from ChIP-qPCR and ChIPseq experiments. Furthermore, MBD2 depletion upon siRNA treatments strongly reduced MBD2 ChIP efficiency on these regions. Thus, the data obtained from ChIPseq experiments performed from antibodies directed against the endogenous proteins seem to be representative of the MBD2 binding profiles of the studied cell lines.

The identification of MBD2 binding sites has provided an opportunity for the search of *in vivo* sequence preferences driving MBD2 deposition. In line with previous biochemical analyses, we did not detect any over-represented sequences in the MBD2 peaks, CG sequence excepted. Nevertheless, the proposed preference for a CCG sequence (77) may escape this analysis since this short sequence is over-represented in CpG islands.

The lack of consensus sequence and the strong enrichment in methylated CpGs at MBD2 peaks support a direct relationship between DNA methylation and MBD2 binding, at least in HMEC-hTERT and HMLER cells. The method used for the mapping of methylated DNA regions was based on the selection of methylated DNA fragments using recombinant proteins associating methylated CpGs. Thus, selected fragments can contain additional CpGs that were not methylated, preventing a direct evaluation of the methylated-CpG density. Nevertheless, combining the mapping of MBD2-tagged proteins with single base pair resolution methylomes allow the establishment of a linear relationship between MBD2 enrichment and methylation density, in neuronal cells (24). It should be noted that, for a minority of MBD2 binding sites, additional events participate in MBD2 deposition. Indeed, we observed that some MBD2 peaks were not associated with methylated DNA regions. In line with this observation, it has been proposed that the NuRD complex mediates the binding of MBD2 to unmethylated DNA regions (24). Altogether, these data indicate that the main determinant for MBD2 binding is the methylation status of the DNA in both HMEC-hTERT and HMLER cells. Furthermore, about 25% of the methylated DNA regions identified from MeDPseq matched MBD2 binding sites. Although we cannot exclude that other methyl DNA binding proteins associated the same methylated DNA regions (82), MBD2 seems to be an important player in the epigenetic machinery in HMEC-hTERT and HMLER cells.

MBD2 knockdown experiments were also in favor of an involvement of MBD2 in the downregulation of many methylated genes. Depletion of MBD2 by siRNA induced an upregulation of about 2,000 genes and 70% of these genes were also upregulated by a DAC treatments in HMLER cells. The analysis of TSS regions of genes upregulated upon MBD2 depletion in untreated HMLER cells indicated that these genes were enriched in MBD2 and DNA methylation marks when compared with genes unaffected or downregulated by MBD2 siRNA. Conversely, the TSS regions of genes upregulated by DAC were enriched in MBD2 when compared with unaffected or downregulated genes upon DNA hypomethylation. The impact of MBD2 depletion on gene expression was globally less stringent than DAC treatments. DNA hypomethylation induced the upregulation of a higher number of genes than MBD2 depletion and higher fold changes in gene expression. It is tempting to explain these differences by the involvement of other MBD proteins and of MBP proteins from other families in the repression of methylated genes. However, the distinct nature of both treatments prevents a quantitative comparison, since data may be biased by differences in treatment efficiencies and kinetics. Further analysis of our data at non-promoter regions indicated that DNA methylation at enhancer sequences was correlated with low transcriptional activity of the genes potentially regulated by these elements, as previously reported in embryonic stem cell (87). Furthermore, a correlation between MBD2 deposition at enhancer sequences and low transcriptional activity was also observed, suggesting that MBD2 may modulate the activity of regulatory regions not directly associated with the 5′ end gene regions. These data also confirm that MBD2 plays an important role in the interpretation of DNA methylation signal in HMLER cells.

In the isogenic cellular model studied, the immortalized cells (HMECT-hTERT) and the oncogenic cells HMLER, most of the MBD2 binding sites were shared by both cell lines. However, oncogenic transformation was associated with a partial redistribution of MBD2 proteins, 5,326 MBD2-positive regions in HMEC-hTERT lost MBD2 binding in HMLER, while 7,419 regions in in HMEC-hTERT gained MBD2 in HMLER cells. Such redistribution may be in part explained by local DNA methylation changes. Nevertheless, the majority of the regions gaining or losing MBD2 in HMLER exhibited comparable MeDPseq-read densities in both cell lines. This redistribution of MBD2 was also observed at promoter regions and associated with the modulation of the transcriptional activity of the corresponding genes. Among the 3,160 genes downregulated in transformed cells, 380 genes were methylated at their promoter regions in both cell lines, specifically associated by MBD2 in HMLER cells, and upregulated upon MBD2 depletion in HMLER. Thus, the redistribution of MBD2 proteins occurring in HMLER cells seemed, at least for a subset of genes, independent of DNA methylation changes. As observed for MeCP2 (76), not all the potential MBD2 binding sites were precipitated by MBD2 antibodies suggesting that additional factors are involved in MBD2 deposition. Several hypotheses, not mutually exclusive, could explain these specific profiles: competition with transcription factors (26), steric hindrance due to chromatin proteins, stabilization of the interaction between MBD2 and methylated DNA by other proteins. Although the precise mechanisms driving the specificity of the MBD family protein profiles remain to be experimentally established, redistribution of MBD2 proteins across methylated DNA regions seems to be associated with the transformation process(es) occurring in the immortalized human mammary cell lines cells expressing the SV40 *T/t* antigens and *H-RAS^V12^*, or either *ZEB*1 or a shP53.

HMLER cells were obtained by sequential introduction of hTERT, SV40 large *T*/small *t* antigens, oncogenic *H-RAS^V12^* genes (33,35) in primary cultures of human mammary epithelial cells. The expression of SV40 sequences affects multiple pathways in human mammary cells (56), raising questions about a SV40-dependent redistribution of MBD2 proteins. We also suppressed the oncosuppressive barriers in HMEC-hTERT cells either by ectopic expression of the transcription factor *ZEB1* or a *P53* depletion. The resulting HMLER cell lines, after introduction of *H-RAS^V12^* gene, exhibited oncogenic properties and a mesenchymal phenotype (36). In HME-ZEB1-RAS and HME-shP53-RAS, most of the 14 regions exhibiting a gain or loss of MBD2 in HMLER cells showed similar MBD2 binding changes. Furthermore, hundreds of genes upregulated by MBD2 depletion in HME-ZEB1-RAS and HME-shP53-RAS cells were also upregulated in HMLER upon treatment with the siRNA targeting *MBD2*. Thus, the dynamics of MBD2 deposition do seem to be independent of the strategy used for overcoming the oncosuppresive barriers. However, we cannot exclude that a large part of the MBD2 redistributions observed are linked to the introduction of *H-RAS^V12^*. MBD2 redistribution and its consequences on gene expression have been investigated in experimental models. Nevertheless, it should be noted that ZEB1 reactivation is a common feature of aggressive and undifferentiated human breast cancers, especially in the claudin-low intrinsic subtype (88), and that alterations of P53-pathways play a crucial role in human cancers (89).

The involvement of MBD2 in the oncogenic process has been already suggested. For example, MBD2 repression of *Lect2* gene may participate in polyp formations via Wnt signaling pathways in mice carrying *Apc^Min^* alleles (29). In human mammary cancer cell lines, the repression of *DAPK1* mediated by MBD2 seems to actively participate in maintenance of their aggressive phenotypes when xenografted into immuno-deficient mice (90). None of these genes was targeted by MBD2 during transformation in the cellular models studied here (Supplementary Table S4). The role of MBD2 in transformation is likely to be due to the repression of multiple targets, which may differ between experimental models or pathologies. Nevertheless, the genes downregulated during the oncogenic transformation of immortalized human mammary cells seem to be preferential targets for MBD2. Furthermore, these genes were preferentially reactivated by MBD2 depletion and enriched in MBD2 binding sites in cell lines constructed from the disruption of oncosuppressive barriers in immortalized cells. It has been reported that in similar cellular models, the major methylation changes at gene promoters occur during the immortalization step, while subsequent steps of oncogenic transformation are associated with subtle modifications of DNA methylation and gene expression (34). In line with these data, we only find minor modifications of the DNA methylation patterns between the immortalized cells, HMEC-hTERT and the oncogenic HMLER cells. Altogether, these data emphasize, at genome-wide level, the role played by the epigenetics readers of DNA methylation during oncogenic transformation. The reprograming of cancer cells may be due to a redistribution of methyl-DNA binding proteins in addition to DNA methylation alterations.

## SUPPLEMENTARY DATA

Supplementary Data are available at NAR Online.

SUPPLEMENTARY DATA
