# Supplementary material for: Dynamics of MBD2 deposition across methylated DNA regions during malignant transformation of human mammary epithelial cells
Source: Nucleic Acids Res. 2015 May 24;43(12):5838–54. doi: 10.1093/nar/gkv508 (PMC4499136; doi:10.1093/nar/gkv508)
Supplement: SUPPLEMENTARY DATA [file supp_43_12_5838__index.html]

Dynamics of MBD2 deposition across methylated DNA regions during malignant transformation of human mammary epithelial cells — Dynamics of MBD2 deposition across methylated DNA regions during malignant transformation of human mammary epithelial cells — SUPPLEMENTARY DATA 

# Dynamics of MBD2 deposition across methylated DNA regions during malignant transformation of human mammary epithelial cells

## SUPPLEMENTARY DATA

- SUPPLEMENTARY DATA
- SUPPLEMENTARY DATA
- SUPPLEMENTARY DATA
- SUPPLEMENTARY DATA
- SUPPLEMENTARY DATA
- SUPPLEMENTARY DATA
- SUPPLEMENTARY DATA
- SUPPLEMENTARY DATA
- SUPPLEMENTARY DATA
- SUPPLEMENTARY DATA
