# Supplementary material for: Dynamics of MBD2 deposition across methylated DNA regions during malignant transformation of human mammary epithelial cells
Source: Nucleic Acids Res. 2015 May 24;43(12):5838–54. doi: 10.1093/nar/gkv508 (PMC4499136; doi:10.1093/nar/gkv508)
Supplement: SUPPLEMENTARY DATA [file supp_gkv508_nar-03334-x-2014-File009.docx]

SUPPLEMENTARY MATERIAL - LEGENDS OF FIGURES AND TABLES.

Figure S1. Comparison between high-throughput sequencing and tiling array technologies. Mean signals from Affymetrix Human Promoter 1.0R tiling arrays were computed at peaks identified from MeDPseq or MBD2 ChIP sequencing experiments. (A-B) MeDP-chip signals, obtained from a recombinant protein containing 4 MBD domains from MBD1, were compared with MeDP sequencing, performed from Invitrogen MethylMiner, in HMEC-hTERT (A) and HMLER (B). (C-D) MBD2 ChIP-chip were compared with MBD2 ChIPseq in HMEC-hTERT (C) and HMLER (D). A.U., Arbitrary Unit.

Figure S2. Examples of MBD2 redistribution during oncogenic transformation. (A) ChIP-qPCR experiments performed with anti-MBD2 antibodies in HMEC-hTERT cells (green) and HMLER cells (red). Each experiment was performed 2 times. (B to O) Genome coverage for MeDP-seq (black), ChIP-MBD2-seq (orange) and Input-seq (gray) in HMEC-hTERT (upper tracks) and HMLER (bottom tracks) at 14 genomic locations tested in A. Scale is in Fragments Per Million (FPM). Blue: Gene. Red: CpG island. Green: qPCR amplicon localization.

Figure S3. Depletion of MBD2 by siRNA strongly decreased MBD2 ChIP efficiency. ChIP-qPCR experiments were performed in HMLER cells treated with a siRNA control (siCtrl) or a siRNA targeting *MBD2* (siMBD2). Each experiment was performed 2 times. (A) Shown regions correspond to the regions analyzed in Figure 2. (B) Shown regions correspond to the regions analyzed in Figure S2.

Figure S4. Transcription at AluY element sites is repressed by MBD2 and DNA methylation. (A) Relative transcription level at AluJ, AluS and AluY element sites in HMLER, as detected by RNA sequencing. HMLER cells were treated with control siRNA (siCtrl), siRNA targeting *MBD2* (siMBD2) or 5-aza-deoxycytidine (DAC). Student t-test relative to siCtrl conditions. * p-value ≤ 0.01. N.S. Non significant. (B) MB2 ChIPseq mean coverages at AluJ, AluS and AluY elements. (C) MeDPseq mean coverages at AluJ, AluS and AluY elements.

Figure S5: DNA methylation and MBD2 binding on CpG island (CGI) cores and shores. (A) MeDPseq coverage at CpG island after clusterisation. (B-C) Basal expression in HMLER (B) and fold change (FC) after 5-aza-deoxycytidine treatment (DAC) (C) for genes with a core methylated CpG island (core), with a shore methylated CpG island (shore) or with an unmethylaled CpG island (unmethylated) within 1kb of their TSS. (D) MBD2 ChIPseq coverage at CpG island after clusterisation. (E-F) Basal expression in HMLER (E) and fold change (FC) after siRNA targeting *MBD2* treatment (F) for genes with a CpG island core bound by MBD2 (core), with a CpG island shore bound by MBD2 (shore) or with an MBD2 unbound CpG island (unbound) within 1kb of their TSS. Wilcoxon tests: N.S. Non significant. * p<0.05. *** p< 0.001

Figure S6. Genes exhibiting MBD2 binding sites or with DNA methylation at their TSS regions have low transcriptional activity. Box-plots showing gene expression levels, determined from RNA-seq, of the total number of genes, genes with MBD2 peaks within +/- 1kb of their TSS, or genes with MeDP peaks within +/- 1kb of their TSS in (A) HMEC-hTERT cells and (B) HMLER cells.

Figure S7. 5-aza-deoxycytidine treatment induced DNA demethylation. Digested and undigested genomic DNA was analyzed on 1% agarose gel. DAC: 5-aza-deoxycytidine. U: undigested. H: digested with the methylation sensitive enzyme HpaII. M: digested with the methylation insensitive enzyme MspI.

Figure S8: DNA methylation and MBD2 binding on enhancers. (A-B) MeDPseq, MBD2 ChIPseq and Input-seq coverage at enhancer after clusterisation. (A) Clusterisation performed on MBD2 ChIPseq read density. (B) Clusterisation performed of MeDPseq read density. (C-E) Basal expression in HMLER (C) and fold change (FC) after an siRNA targeting MBD2 treatment (D) or a 5-aza-deoxycytidine (DAC) treatment (E) for all genes (1), for genes associated with a MBD2-bound enhancer (2) or for genes associated with a methylated enhancer (3) as identified in A and B. Wilcoxon tests: * p<0.05. ** p<0.01 *** p< 0.001

Figure S9. Expression levels of genes gaining MBD2 at their TSS regions in HMLER, HME-ZEB1-RAS, and HME-shP53-RAS cells compared with HMEC-hTERT cells (see Figure S2 and Figure 7B). For each gene, relative values were determined using 1 for the expression level in HMEC-hTERT cells. (A) Green: Expression level in HMEC-hTERT. Red: Expression level in HMLER treated with a control siRNA. Orange: Expression level in HMLER treated with siRNA against *MBD2*. Black: Expression level in HMLER treated with DAC (5-aza-deoxycytidine). Error bars from duplicate RNAseq experiments. (B) Green: Expression level in HMEC-hTERT. Blue: Expression level in HME-ZEB1-RAS treated with a control siRNA. Purple: Expression level in HME-shP53-RAS treated with a control siRNA. Orange: Expression level in HME-ZEB1-RAS or HME-shP53-RAS treated with a siRNA against *MBD2*. Error bars from duplicate RNAseq experiments.

Figure S10: Gain of MBD2 binding near the transcription start sites of 247 genes, repressed during oncogenic transformation, and upregulated by a siMBD2 treatment in at least two of the three studied transformed cell lines (see figure 6C and 6D).

Supplementary Table 1. Sequences and hybridization temperatures of primers used in qPCR experiments.

Supplementary Table 2. DNA methylation analysis by next generation bisulfite sequencing. DNA methylation patterns of 9 genes were determined by parallel sequencing of PCR fragments obtained from bisulfite modified DNA. For each gene and cell line, duplicates were performed from two independent experiments. HMLER cells were treated with 10 μM of DAC for 72 hours. “Start and end” indicated the genomic position of the PCR fragments, using hg19 as a genomic reference.

Supplementary Table 3. Analysis of enriched K-mers sequences in MBD2 peaks in HMEC-hTERT and HMLER. Genomic sequences of MBD2 peaks were analyzed using RSAT oligo-diff tools (50).

Supplementary Table 4. RNAseq Reads Per Million (RPM) values for all genes in HMEC-hTERT, HMLER treated with siCtrl, siMBD2 or DAC, HME-ZEB1-RAS treated with siCtrl or siMBD2, HME-shP53-RAS treated with siCtrl or siMBD2. For each experiment duplicates are reported in the table.

Supplementary Table 5. Top 15 GO terms or KEGG pathways enriched in genes upregulated upon MBD2 depletion in HMLER, HME-ZEB1-RAS or HME-shP53-RAS (GSEA analysis). NES: Normalized Enrichment Scores. FDR: False Discovery Rate.
