# Supplementary material for: Dynamics of MBD2 deposition across methylated DNA regions during malignant transformation of human mammary epithelial cells
Source: Nucleic Acids Res. 2015 May 24;43(12):5838–54. doi: 10.1093/nar/gkv508 (PMC4499136; doi:10.1093/nar/gkv508)
Supplement: SUPPLEMENTARY DATA [file supp_gkv508_nar-03334-x-2014-File010.pdf]

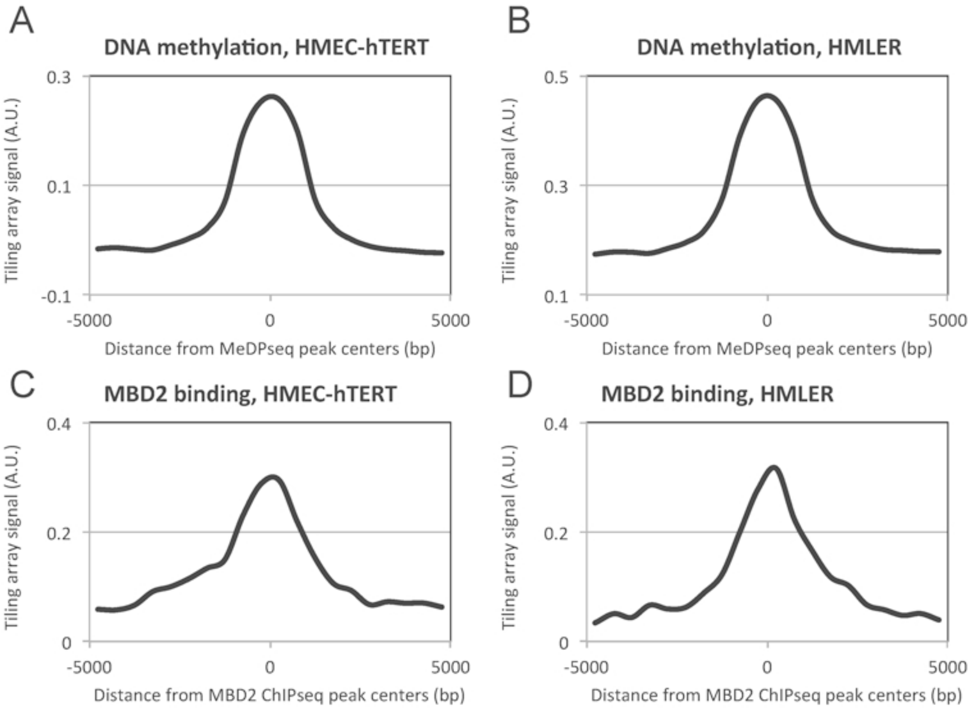

Fig S1 Devailly et al.

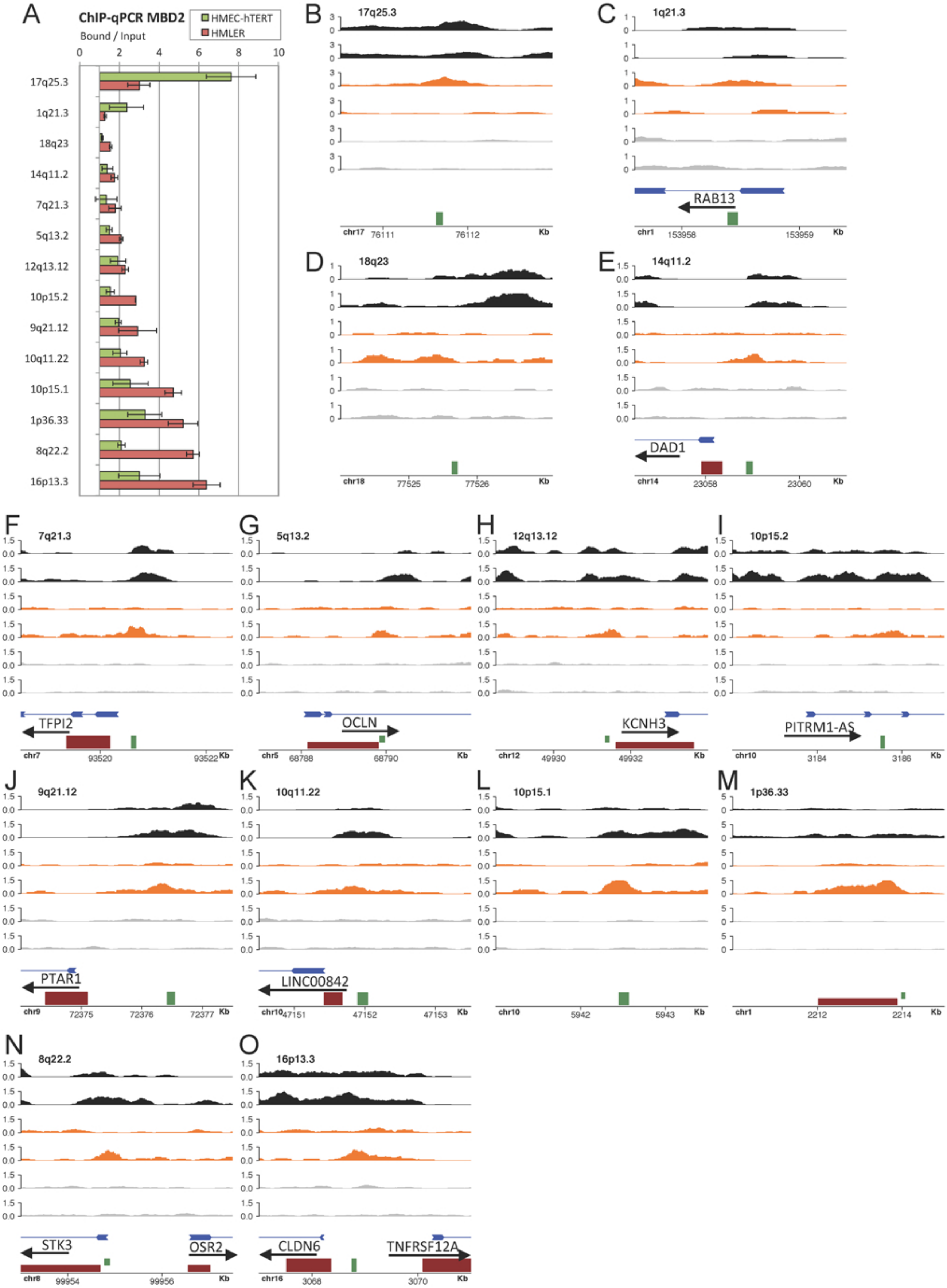

Fig S2 Devailly et al.

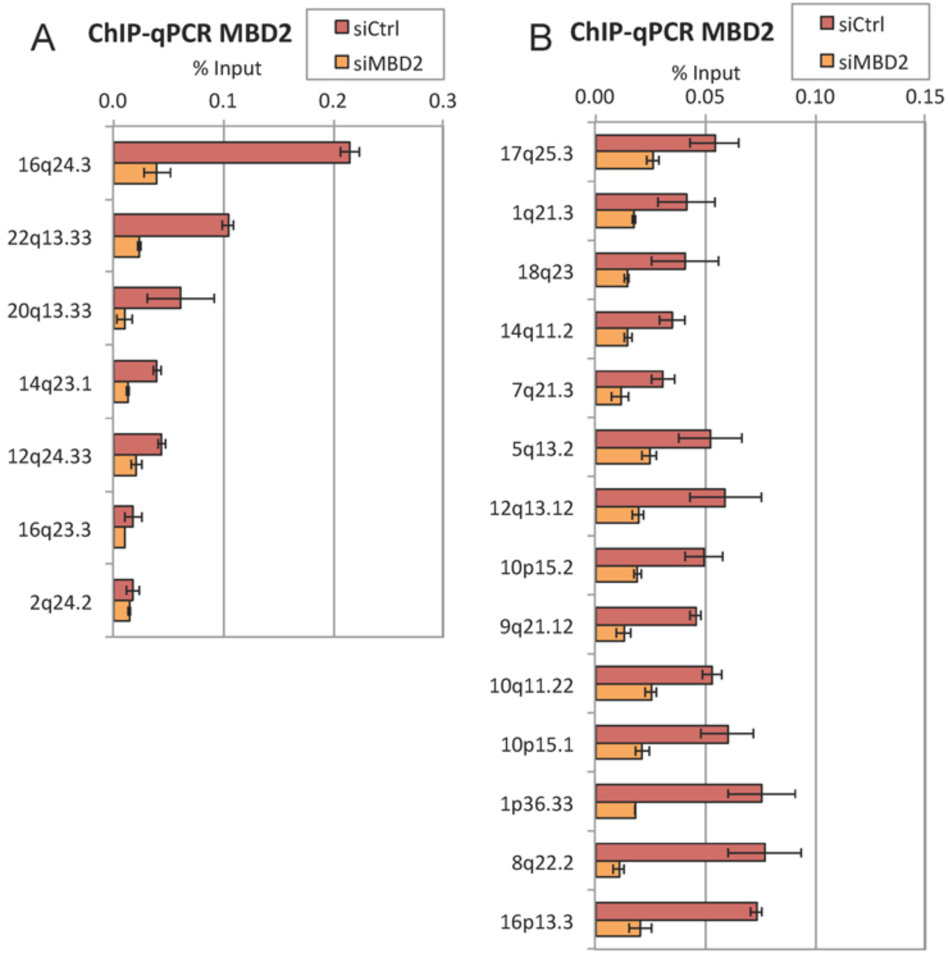

Fig S3 Devailly et al.

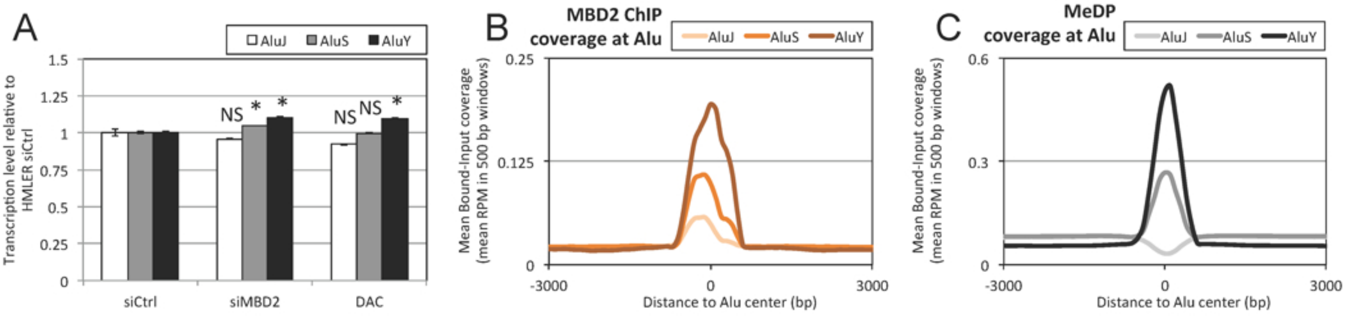

Fig S4 Devailly et al.

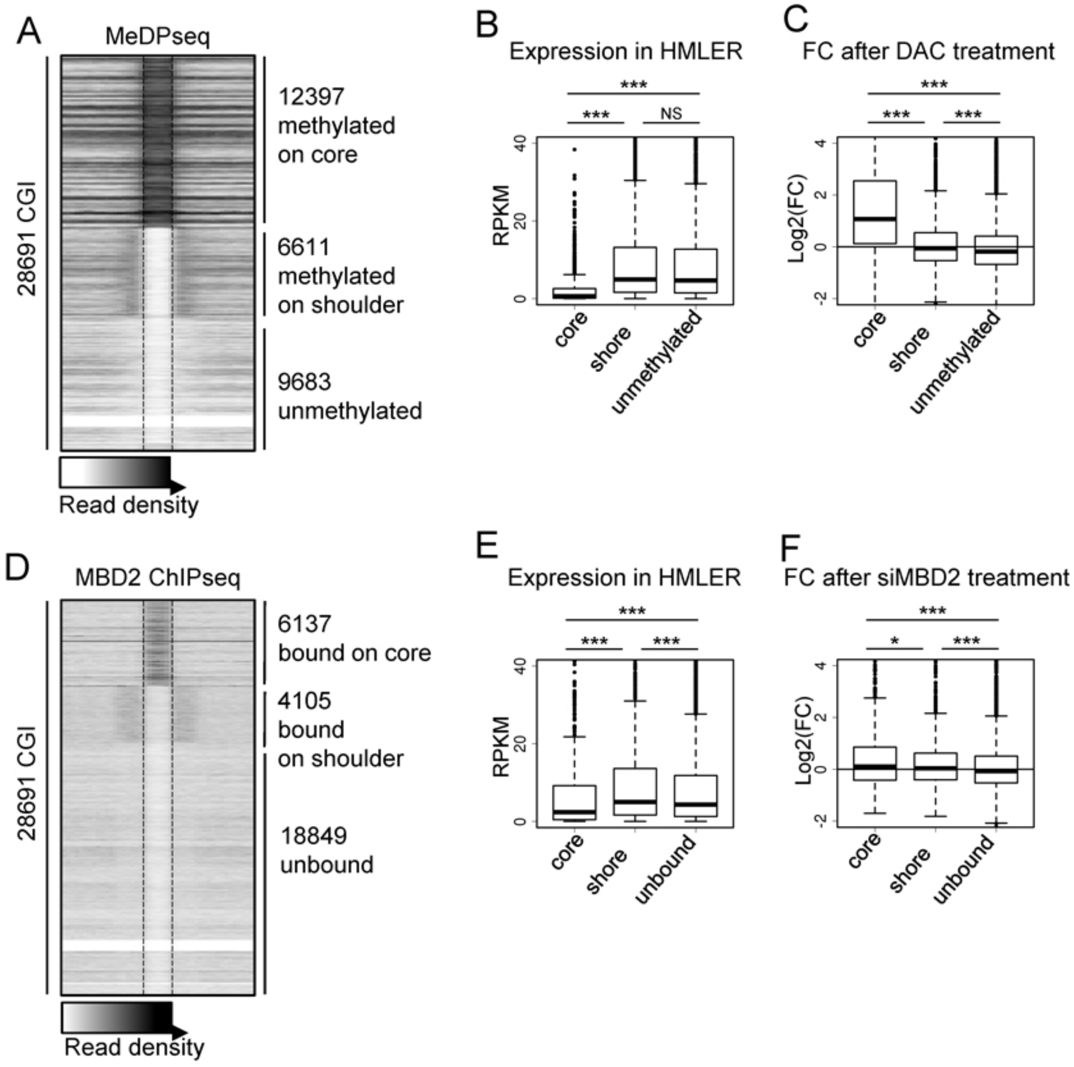

Fig S5 Devailly et al.

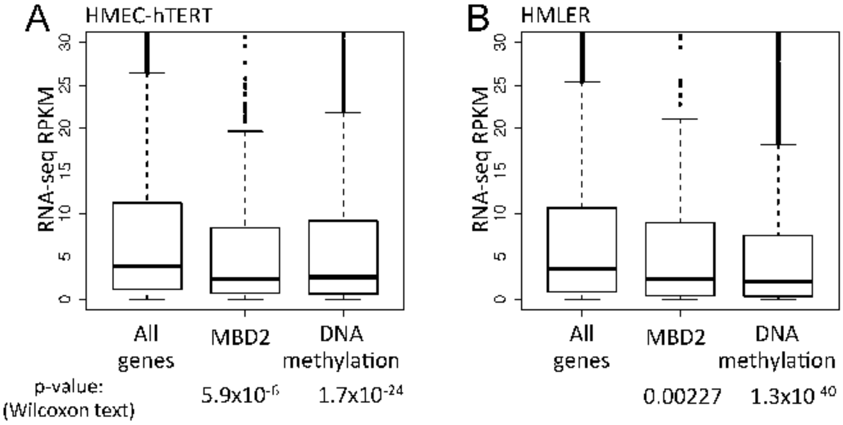

Fig S6 Devailly et al.

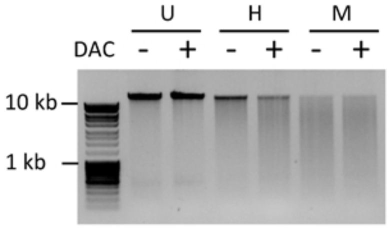

Fig S7 Devailly et al.

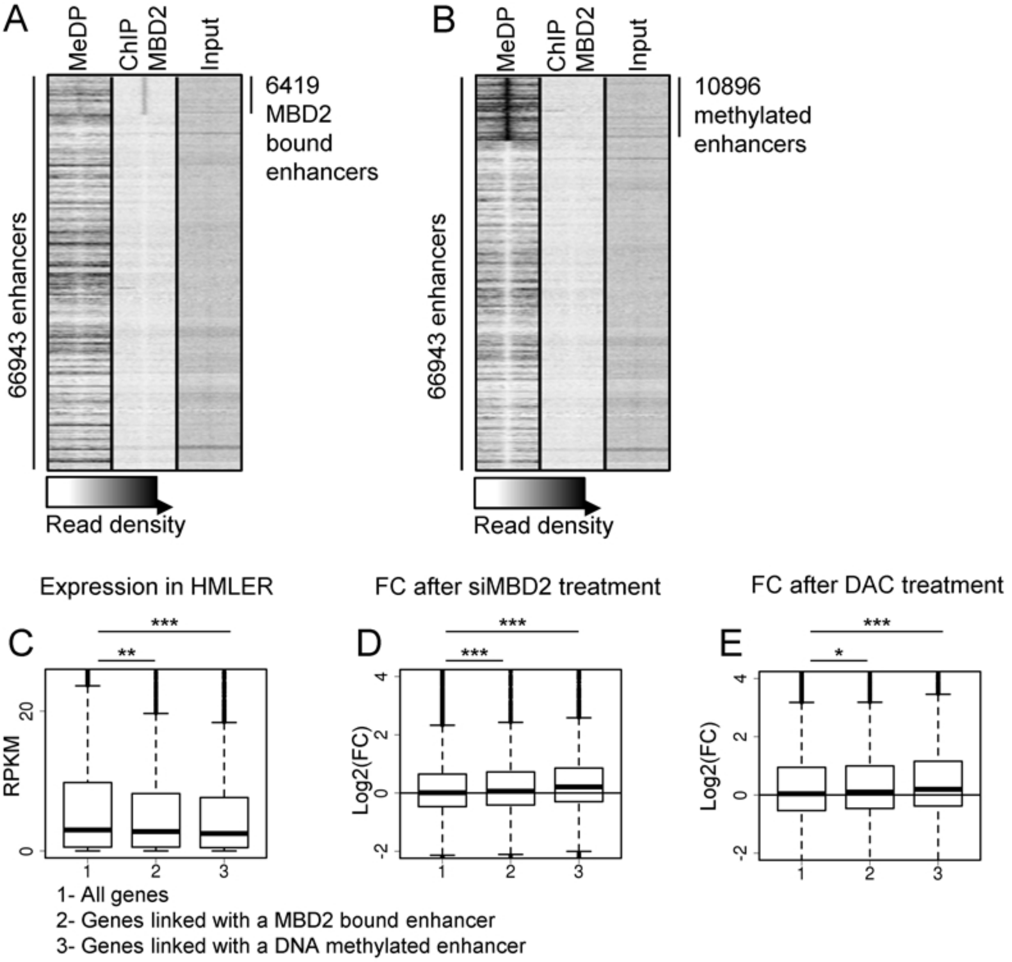

Fig S8 Devailly et al.

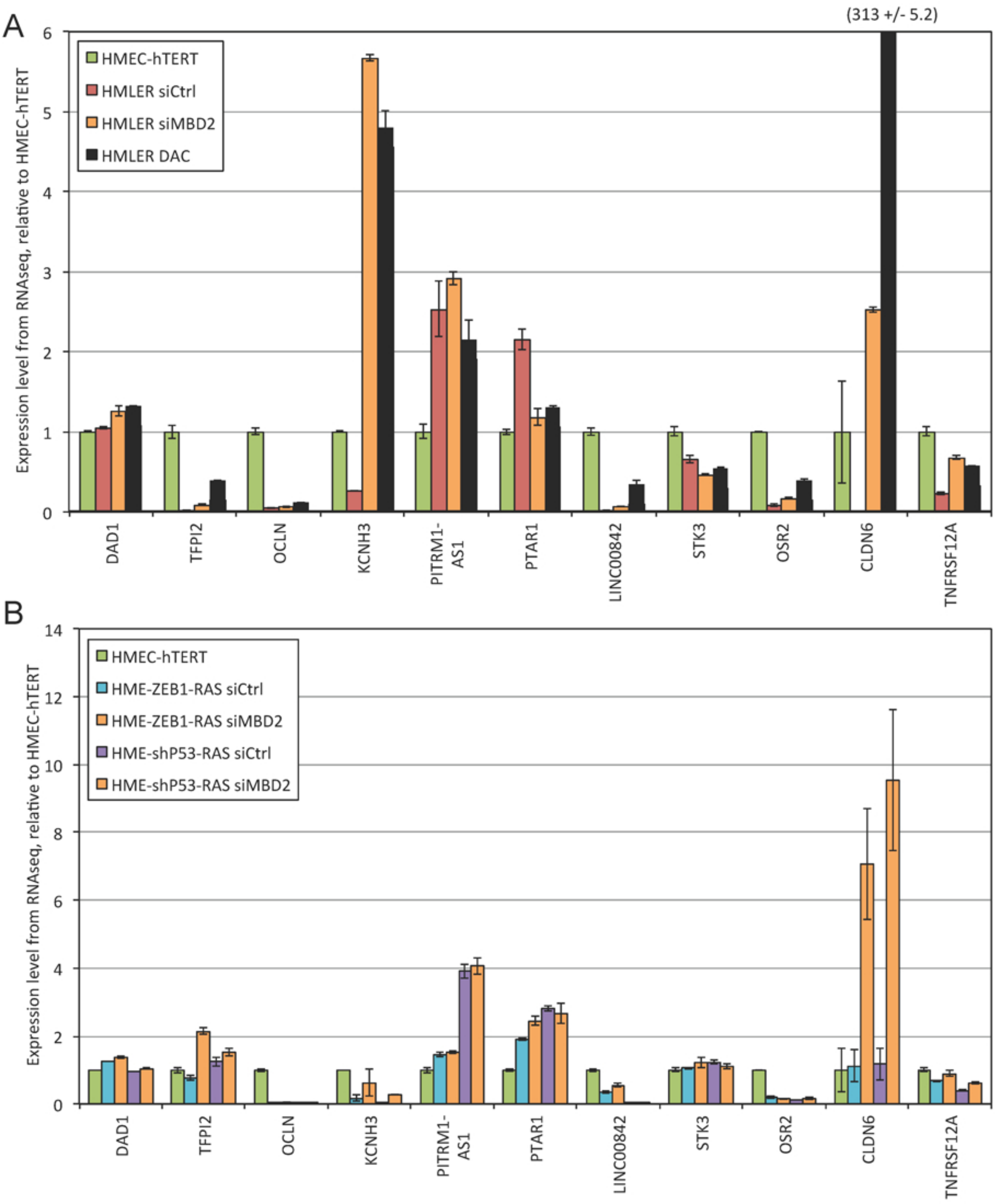

Fig S9 Devailly et al.

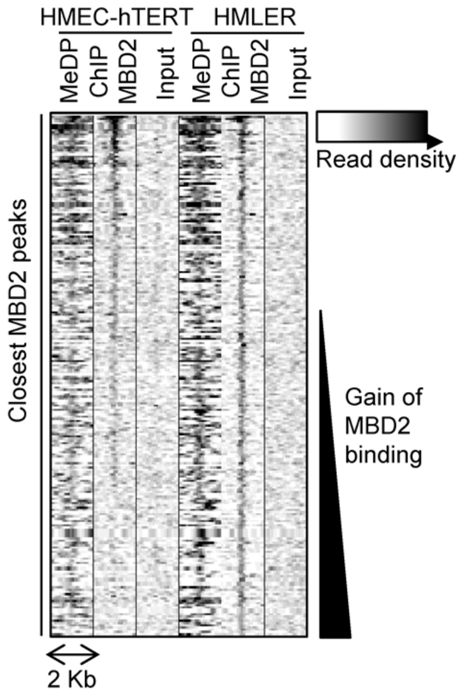

Fig S10 Devailly et al.

Figure S10: Gain of MBD2 binding near the transcription start sites of 247 genes, repressed during oncogenic transformation, and upregulated by a siMBD2 treatment in at least two of the three studied transformed cell lines.
